# Paleoceanography of the northwestern Pacific across the Early–Middle Pleistocene boundary (Marine Isotope Stages 20–18)

**DOI:** 10.1186/s40645-020-00395-3

**Published:** 2021-04-30

**Authors:** Yoshimi Kubota, Yuki Haneda, Koji Kameo, Takuya Itaki, Hiroki Hayashi, Kizuku Shikoku, Kentaro Izumi, Martin J. Head, Yusuke Suganuma, Makoto Okada

**Affiliations:** 1 Department of Geology and Paleontology, National Museum of Nature and Science, 4-1-1, Amakubo, Tsukuba, Ibaraki, 305-0005 Japan; 2 Geological Survey of Japan, National Institute of Advanced Industrial Science and Technology, Tsukuba Central 7, 1-1-1 Higashi, Tsukuba, Ibaraki, 305-8567 Japan; 3 Department of Earth Sciences, Faculty of Science, Chiba University, 1-33, Yayoi, Inage, Chiba, Chiba 263-8522 Japan; 4 Interdisciplinary Graduate School of Science and Engineering, Shimane University, Nishikawatsu-Cho 1060, Matsue, Shimane 690-8504 Japan; 5 Faculty & Graduate School of Education, Chiba University, 1-33 Yayoi-Cho, Inage-Ku, Chiba, Chiba 263-8522 Japan; 6 Department of Earth Sciences, Brock University, 1812 Sir Isaac Brock Way, St. Catharines, ON L2S 3A1 Canada; 7 National Institute of Polar Research, 10-3, Midori-Cho, Tachikawa, Tokyo, 190-8518 Japan; 8 Department of Polar Science, School of Multidisciplinary Sciences, The Graduate University for Advanced Studies (SOKENDAI), Hayama, Japan; 9 Department of Earth Sciences, Ibaraki University, 2-2-1, Bunkyo, Mito, Ibaraki, 310-8512 Japan

## Abstract

The fluctuating position of the boundary between the Kuroshio (warm) and Oyashio (cold) currents in the mid-latitude western North Pacific affects both heat transport and air–ocean interactions and has significant consequences for the East Asian climate. We reconstruct the paleoceanography of Marine Isotope Stages (MIS) 20–18, MIS 19 being one of the closest astronomical analogues to the present interglacial, through multiple proxies including microfossil assemblage data, planktonic foraminiferal isotopes (δ^18^O and δ^13^C), and foraminiferal Mg/Ca-based temperature records, from the Chiba composite section (CbCS) exposed on the Boso Peninsula, east-central Japan. Principal component analysis (PCA) is used to capture dominant patterns of the temporal variation in these marine records, and shows that the relative abundances of calcareous nannofossil and radiolarian taxa are consistent with the water mass types inferred from geochemical proxies. The leading mode (36.3% of total variance) mirrors variation in the terrestrial East Asian winter monsoon (EAWM), reflecting seasonal trends dominated by the winter monsoon system. In the CbCS, this mode is interpreted as reflecting the interplay between the warm Kuroshio and cold Oyashio waters, which is likely related to the latitudinal shift of the subtropical–subarctic gyre boundary in the North Pacific. The second mode (15.4% of total variance) is closely related to subsurface conditions. The leading mode indicates that MIS 19b and 19a are represented by millennial-scale stadial/interstadial oscillations. Northerly positions for the gyre boundary during late MIS 19c, the interstadials of MIS 19a, and early MIS 18 are inferred from the leading mode, which is consistent with a weak EAWM and consequent mild winter climate in East Asia. Nonetheless, the northerly positions for the gyre boundary during late MIS 19c and early MIS 19a were not associated with subsurface warming presumably due to the suppressed gyre circulation itself caused by the weak Aleutian Low. Intermittent southerly positions for the gyre boundary are inferred for the stadials of MIS 19b and 19a. Regional sea surface temperature (SST) comparisons in the western North Pacific reveal that the moderate SSTs during MIS 19a through early MIS 18 were restricted to the mid- to high latitudes, influenced by the weak EAWM. Comparison between MIS 20–18 and MIS 2–1 suggests that glacial MIS 20 and 18 had significantly milder winters than MIS 2, likely related to the relatively weak EAWM.

**Supplementary Information:**

The online version contains supplementary material available at 10.1186/s40645-020-00395-3.

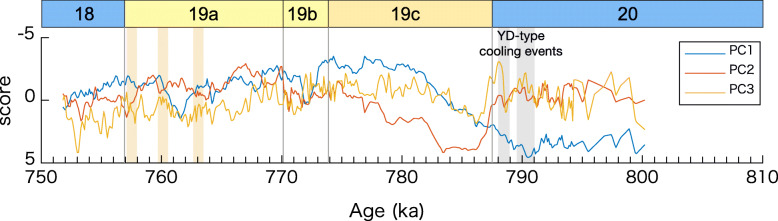

## Introduction

Pronounced glacial–interglacial oscillations characterize the Quaternary Period. The Early–Middle Pleistocene transition (EMPT) spanning ~ 1.4–0.4 Ma (Ruddiman et al. 1986; Head and Gibbard 2015a) saw a fundamental and progressive shift from 41 ky to quasi-100 ky cyclicity along with an increase in global ice volume during glacials and a decrease in the relative duration of interglacials compared to glacial stages (Lisiecki and Raymo 2007). Orbital controls on Earth’s climate, namely precession, obliquity, and eccentricity, change latitudinal and seasonal insolation and have modulated the climate system throughout the Quaternary (Cronin 2010). Marine Isotope Stage (MIS) 19, occurring at ~ 790–761 ka within the middle of the EMPT, along with MIS 11 (424–374 ka), are the closest analogues for the present interglacial in terms of astronomical and paleoclimatic signatures (Tzedakis et al. 2009; Yin and Berger 2011; Tzedakis et al. 2012; Head and Gibbard 2015a; Head in revision). Therefore, paleoceanographic records for MIS 19 offer a crucial understanding of the natural variability of our modern climate in the absence of anthropogenic CO_2_ emissions, and so provide a baseline needed to predict future climate change.

The early phase of the EMPT (~ 1.4–0.7 Ma) is characterized by increasingly pronounced global and regional climate changes such as severe glaciations (e.g., Clark et al. 2006; Elderfield et al. 2010; Head and Gibbard 2015a), evolution of the tropical sea surface temperature (SST) pattern (de Garidel-Thoron et al. 2005; Dyez and Ravelo 2014), and changes in the Asian monsoon and vegetation (Sun et al. 2006, 2019). A reconstruction of SSTs in the tropical Pacific inferred that the zonal east–west SST contrast increased during ~ 1.2–0.8 Ma, resulting in a shift from El Niño-like to more La Niña-like conditions (de Garidel-Thoron et al. 2005; Dyez and Ravelo 2014). In Asia, a series of studies on the Chinese Loess Plateau revealed that the amplitude and cyclicity of the Asian monsoon and vegetation had changed during the ~ 1.2–0.7 Ma interval (Sun et al. 2006, 2019). This evidence suggests that developments during the early EMPT involved dynamic circulation changes including an intensification of the Walker circulation and trade winds (Dyez and Ravelo 2014) and probably enhancement of the Asian monsoon system (Han et al. 2012).

The Kuroshio is a north flowing boundary current in the western North Pacific and represents one of the Earth’s critical heat engines, playing a crucial role in transporting heat poleward (Talley 2013). The Oyashio, another North Pacific western boundary current but in the subarctic gyre, carries abundant nutrients and its fluctuations impact primary production along the coast of the northern Japanese archipelago. The evolution of these two current systems is therefore of significant interest. Marine records of calcareous microfossils are sparse for the western North Pacific where their preservation is often poor owing to the shallow carbonate compensation depth. On the other hand, long-term environmental changes during the early EMPT have been investigated using diatoms (Sancetta and Silvestri 1986) and radiolarians (Matsuzaki et al. 2015), which are relatively well preserved in the North Pacific, although the temporal resolution of these studies is low. The early EMPT in the western North Pacific (~ 1.3–0.7 Ma) is characterized by warm and oligotrophic conditions based on the radiolarian fauna (Matsuzaki et al. 2015), suggesting the northward migration of the Kuroshio Current probably in response to the intensification of the East Asian summer monsoon and associated enhanced trade winds (Han et al. 2012; Matsuzaki et al. 2015). Together with the strong Kuroshio Current during the early EMPT, the radiolarian record suggests that the subarctic Oyashio Current migrated southward (Matsuzaki et al. 2015), which is consistent with enhanced cold conditions in the higher latitudes of the North Pacific (Sancetta and Silvestri 1986; Morley and Dworetzky 1991; Matul et al. 2009; Zhang et al. 2014). However, it remains unclear how these environmental changes were linked to the regional-scale re-organization of the circulation system (e.g., Walker Circulation and Asian monsoon systems) due to the lack of high-resolution data.

The Chiba composite section (CbCS) including the Chiba section itself (35° 17′ 39.6"N, 140° 08′ 47.6" E to 35° 17′ 36.9" N, 140° 08′ 47.2" E; Suganuma et al. 2021) exposed on the Boso Peninsula, central Japan, and occurring within the Kokumoto Formation of the Kazusa Group (Kazaoka et al. 2015), represents a high-resolution continuous sedimentary succession deposited on the shelf edge to continental slope at depths greater than 200 m (Nishida et al. 2016). This succession contains excellently preserved calcareous microfossils (Kameo et al. 2020; Suganuma et al. 2018), and its favorable nature has permitted the establishment of a centennial- to millennial-scale benthic foraminiferal oxygen isotope (δ^18^O) stratigraphy (Okada et al. 2017; Suganuma et al. 2018; Haneda et al. 2020a). The boundary between the warm Kuroshio and cold Oyashio currents is presently situated just east of the Boso Peninsula (Qiu 2001). During the late Quaternary, the position of the gyre boundary between the subtropical Kuroshio and subarctic Oyashio currents was oscillating at orbital (Moore et al. 1980; Thompson and Shackleton 1980; Chinzei et al. 1987; Oba et al. 2006) and millennial (Yamamoto et al. 2005) time scales, causing significant changes in SST and nutrient levels in the mixed zone (Oba et al. 2006; Yamamoto et al. 2005). Multiple paleoceanographic proxies obtained from the CbCS should, therefore, provide insights into the Kuroshio and Oyashio currents variability during a significant interval (MIS 20–18) at an ideal location.

The CbCS unambiguously records the Matuyama–Brunhes (M–B) paleomagnetic polarity boundary (Suganuma et al. 2015) which is the primary chronological datum for the Lower–Middle Pleistocene Subseries boundary (Head et al. 2008; Head and Gibbard 2015b; Gibbard and Head 2020). The boundary occurs immediately above the regionally widespread and prominent Ontake-Byakubi-E (Byk-E) tephra bed (Suganuma et al. 2015; Hyodo et al. 2016; Takeshita et al. 2016; Okada et al. 2017; Haneda et al. 2020b). The combination of the M–B boundary, the marine isotope stratigraphy, and the Byk-E tephra bed allows paleoenvironmental proxies within the CbCS to be correlated at local, regional, and global scales. The Global boundary Stratotype Section and Point for the Middle Pleistocene Subseries and Chibanian Stage is placed at the base of the Byk-E tephra bed at the Chiba section (Head, 2019; Suganuma et al. 2021).

In this study, we synthesize paleoceanographic records of the CbCS, including microfossil fauna and flora, foraminiferal isotope (δ^18^O and δ^13^C), and Mg/Ca-based temperature records, to achieve an objective understanding of marine paleoenvironmental changes characterizing the late MIS 20 – early MIS 18 interval. We then compare the CbCS records with published SST reconstructions at lower and higher latitudes in the western North Pacific and with terrestrial records in East Asia, to explore paleoenvironmental links between the CbCS and regional to global climate oscillations. In addition to the published CbCS data in Okada et al. (2017), Suganuma et al. (2018), and Haneda et al. (2020a), we newly report on an Mg/Ca-based temperature record for *Globigerina bulloides* and on high-resolution δ^13^C data for *G*. *bulloides* and *Globorotalia inflata*.

### Modern oceanographic setting

**Fig. 1:**
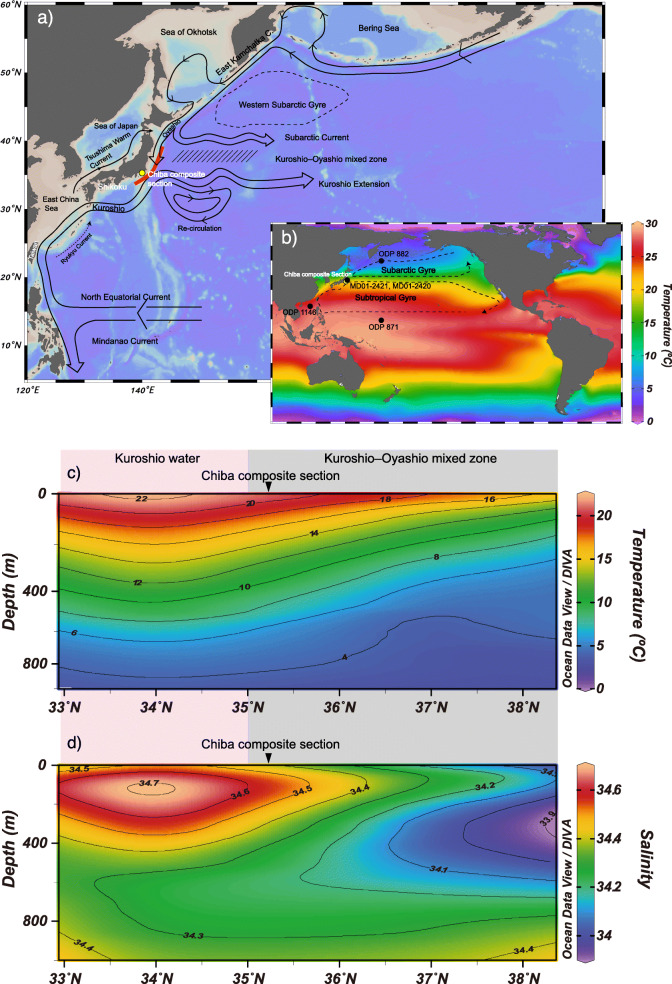
The current system in the western North Pacific and latitudinal water properties, showing **a** regional current systems (Qiu 2001) and the location of the Chiba composite section (CbCS) **b** the two North Pacific gyres based on Qiu (2001) and core site locations. Latitudinal **c** temperature (Locarnini et al. 2013), **d** salinity (Zweng et al. 2013) profiles in the Kuroshio and Kuroshio–Oyashio mixed zone obtained from the red line in panel **a**

The North Pacific is largely divided into two main circulatory systems, the subtropical and subarctic gyres (Qiu 2001) (Fig. 1). The subtropical gyre is maintained by the trade winds in the tropics and the westerlies at mid-latitudes (Qiu 2001). The trade winds drive equatorial surface current systems westward in the tropical Pacific, resulting in a large warm area (> 28 °C) distributed in the western tropical Pacific (De Deckker 2016). Known as the western Pacific Warm Pool (WPWP), it serves as the heat engine of the globe (De Deckker 2016).

The Kuroshio Current branches from the North Equatorial Current generally at 12–13° N off the Philippines in the northern part of the WPWP, flowing northwards where it enters the East China Sea from the east of Taiwan (Qiu 2001). The Kuroshio Current, therefore, transports a large quantity of heat and salt from the WPWP to the mid-latitude western North Pacific. Further downstream, the Kuroshio Current enters the deep Shikoku Basin from the East China Sea through the Tokara Strait and flows northeastward until 140° E and 35° N (Qiu 2001). After diverging from the Japanese coast, the Kuroshio Current turns eastward and is renamed the Kuroshio Extension (Qiu 2001).

Regarding seasonal variations for the Kuroshio Current, volume transport is typically higher in July/August and is reduced in October in the East China Sea (Kawabe 1988; Andres et al. 2008). A branch of the Kuroshio Current, the Ryukyu Current, a subsurface-intensified current, flows on the forearc side of the Ryukyu Islands, although its mean volume transport is much less than for the Kuroshio Current, and has large temporal and spatial variability (Andres et al. 2008, 2015). The Kuroshio Current strengthens from a mean downstream transport of about 15 Sv east of Luzon to 24 ± 0.9 Sv in the East China Sea (Andres et al. 2015). The Kuroshio further strengthens to 65 ± 6 Sv off Shikoku, fed in part by the Ryukyu Current (Zhu et al. 2003; Andres et al. 2015). South of Japan, the Kuroshio path meanders with a 2–10 year cyclicity (Kawabe 1995). Seasonal variation in the Kuroshio volume transport is highly variable south of Japan (~ 133.5° E), varying with the year of observation (Book 2002; Kakinoki et al. 2008). Interannual to decadal variation in the Kuroshio Current's volume transport is controlled by wind stress curl over the Pacific (Andres et al. 2011). Wind stress curl is induced by the trade winds and westerlies over the central North Pacific, and Rossby waves propagate variations in the wind stress far westward (Deser et al. 1999). The clockwise wind stress curl is related to the strength of the Aleutian Low (AL) and westerlies on a decadal timescale (Hanawa and Kamada 2001).

Water masses of the Kuroshio Current are characterized by a salinity maximum at depths of 100–250 m and a salinity minimum at 500–700 m, with the main thermocline occurring between them (Fig. 1c and d; Nitani 1972; Masuzawa 1972). The water in the salinity maximum zone originates from North Pacific Tropical Water (NPTW), formed at the sea surface by strong evaporation at 20–30° N in the central region of the North Pacific (Oka and Kawabe 1998; Katsura et al. 2013). NPTW subducts from the surface to a depth of 200 m in the southern part of the subtropical gyre, and is then advected westward by the North Equatorial Current and poleward by the Kuroshio (Tsuchiya 1968). Variations in the NPTW are linked to wind strength over the subtropical gyre (Shuto 1996), with wind intensification leading to an increase in the transport of NPTW (Suga et al. 2000). Remnants of NPTW in the Kuroshio are recognizable as a subsurface salinity maximum and presumably contribute to the formation of the broad subsurface salinity maximum that is typical in the northwestern part of the subtropical gyre (Suga et al. 2000).

The subarctic gyre in the North Pacific drives another western boundary current, the Oyashio Current (Qiu 2001). The Oyashio Current originates from the East Kamchatka Current in the Sea of Okhotsk but has different water properties including high levels of dissolved oxygen in the upper 700 m. The total volume transport of the Oyashio reaches 20–30 Sv in winter and spring and decreases to 3–4 Sv in summer and fall, which is consistent with the annual rhythm of the wind-driven subtropical gyre. The Oyashio branches into two paths: one turns east at 42° N southeast of Hokkaido, while the other continues southward along the margin of Japan as far as 38.7° N. Below the surface of the Oyashio Current, North Pacific Intermediate Water (NPIW) extends to intermediate depths (300–800 m) that are well-defined by a salinity minimum (e.g., Talley 1993; Yasuda 1997; Yasuda 2004). The NPIW originates from the Okhotsk Sea Intermediate Water (~ 200–1000 m deep), which is characterized by low temperatures (1.0–1.5 °C), low-salinity (33.5–33.7), and high oxygen content (3.3 to 4.9 mL/L) (Itoh et al. 2003). The Okhotsk Sea Intermediate Water is formed by Western Subarctic Pacific Water that is modified by surface cooling and freshening in the Okhotsk Sea and dense shelf water produced by brine rejection during the growth of sea ice along the northern shelves of the Okhotsk Sea (Yamamoto et al. 2002). The NPIW is mainly formed along the Kuroshio Extension (Hiroe et al. 2002) and possesses a salinity range of 33.9–34.1 (Bostock et al. 2010). The low salinity NPIW reflects the dominance of precipitation in the subpolar North Pacific. Thus, it can be regarded as the low-salinity counterpart of the high salinity NPTW (Suga et al. 2000).

At present, the Kuroshio Current’s warm and saline water marginally reaches the CbCS (Fig. 1a–d). The Kuroshio and Oyashio currents mix in the area between 35 and 42° N, forming steep latitudinal SST and sea surface salinity (SSS) gradients (Fig. 1c and d). The annual SST in the Kuroshio region (~ 34° N) is ~ 22 °C, dropping to 16 °C in the Kuroshio–Oyashio mixed zone (~ 38° N) (Fig. 1b and c). Similarly, annual SSS in the Kuroshio region is 34.5, whereas in the mixed zone it is 34.1 (Fig. 1d). Accordingly, sea surface oxygen isotopes (δ^18^O_w_) indicate + 0.1 to − 0.2‰ (VSMOW) in the Kuroshio region, and − 0.75‰ (VSMOW) near the Oyashio Front (~ 40° N) (δ^18^O_w_ = 0.521, SSS = − 17.955; Oba et al. 2006). These SST and SSS latitudinal gradients affect foraminiferal calcite δ^18^O in the Kuroshio–Oyashio mixed zone as follows. Foraminiferal δ^18^O changes by + 0.32‰/°N (− 1.5 °C/°N) due to the effect of SST (− 0.21‰ °C^−1^, Bemis et al. 1998) and only by − 0.05‰/°N (− 0.1 in salinity/°N) due to SSS (Oba et al. 2006) between 34° N and 38° N (Fig. 1c and d).

AL activity during the winter dominantly impacts upper oceanic states in the North Pacific (Sugimoto and Hanawa 2009). Variations in SSTs in the Kuroshio–Oyashio mixed zone and the magnitude of the subtropical and subpolar gyres are primarily caused by AL activity (Latif and Barnett 1996; Ishi and Hanawa 2005; Yasuda and Sakurai 2006). When the location of the AL shifts to the east (west) in its strengthening (weakening) phase, westerlies strengthen (weaken) in association with an enhancement (diminishment) of both subtropical and subarctic gyres (Sugimoto and Hanawa 2009). This longitudinal shift occurs on an interdecadal timescale, whereas the latitudinal change of the AL shifts on a timescale of about ten years (Sugimoto and Hanawa 2009). The latitudinal shift is independent of the variation in intensity of the AL and causes a meridional shift of the gyre boundary (Sugimoto and Hanawa 2009). Accordingly, SSTs in the Kuroshio–Oyashio mixed zone and Kuroshio Extension area change in association with the latitudinal shift of the AL and the westerlies (Sugimoto and Hanawa 2009). An enhanced AL is tightly coupled with an intensified East Asian winter monsoon (EAWM) (Chang et al. 2006). On an interannual scale, the EAWM tends to be weaker in El Niño years and stronger in La Niña years (e.g., Zhang et al. 1996).

In terms of nutrients, Kuroshio water is relatively oligotrophic resulting in low biological productivity in the downwelling-dominant subtropical gyre, whereas the Oyashio is rich in nutrients fed by the upwelling-dominated subarctic gyre in the north (Qiu 2001). Therefore, nutrient levels in the upper part of the water column in the Kuroshio–Oyashio mixed zone are controlled by either water mass advected into the area.

## Methods

### Chiba composite section and age model

The Chiba composite section (CbCS) comprises the Urajiro, Yanagawa, Yoro River, Yoro-Tabuchi, and Kokusabata sections (Suganuma et al. 2021). We used the age model of Suganuma et al. (2018), this being the latest age model and based on the composite benthic δ^18^O stratigraphy astronomically tuned to the sea level proxy curve of Ocean Drilling Program (ODP) Site 1123 off New Zealand (Elderfield et al. 2012). This chronology is supported by the U-Pb radiometric age of the Byk-E tephra (Suganuma et al. 2015). The age model uncertainty is thought to be ca. 5 ka as inferred from the chronologic uncertainty of 4 ka in the LR04 stack used as a target curve by Elderfield et al. (2012) (Lisiecki and Raymo 2005) plus another ca. 1 ka of uncertainty in our tuning to the Elderfield et al. (2012) record (Suganuma et al. 2021).

### Carbon isotope analysis

We here present a high temporal resolution δ^13^C record for planktonic foraminifers *G*. *bulloides* and *G*. *inflata* from the CbCS, which was analyzed together with the already published δ^18^O records in Okada et al. (2017), Suganuma et al. (2018), and Haneda et al. (2020a). The temporal resolution of the composite data set is 170 years for *G. bulloides* (*n* = 309) and 160 years for *G.* *inflata* (*n* = 336). The sampling interval is 35 cm on average. The horizons analyzed are listed in Additional files 1 and 3.

The samples, all siltstones, were disaggregated with sodium sulfate and/or using a SELFRAG high voltage pulse fragmentation system installed at the National Institute of Polar Research (NIPR) in Tokyo, Japan. The non-magnetic fraction, including foraminifers, was then extracted from the siltstone samples using an isodynamic separator at Ibaraki University. We manually picked foraminiferal tests from the non-magnetic fraction for each sample. For each sample, where possible, we selected more than 20 individuals of *G*. *inflata* with a test diameter greater than 250 μm for the δ^13^C analysis. We used *G*. *bulloides* from the > 250 μm size fraction for the Yanagawa (sample ID: YN and YG), Urajiro (sample ID: YW), and Kokusabata (sample ID: KG) sections, and 150–250 μm size fraction for the Yoro River (sample ID: TB and YT) and Yoro-Tabuchi (sample ID: TB2) sections due to the low abundance of this species (Additional file 1). It was occasionally necessary to use fewer than 20 individuals in samples where abundances of this species were low.

The preservation of foraminifers was generally good to excellent with very slight fragmentation (Suganuma et al. 2018). Preservation was assessed under the stereomicroscope and SEM (Pearson and Burgess 2008). The foraminiferal tests were translucent and “glassy” under stereomicroscopy except for thickly crusted *G*. *inflata*, indicating no apparent diagenetic features (i.e., dissolution, overgrowth, and recrystallization). The finer-scale structures of typical foraminiferal tests for *G*. *bulloides* were observed under SEM (Additional file 4). The SEM photographs indicate that the surfaces are slightly rough in some parts but microstructures such as the spine bases are well preserved. The roughened surfaces probably represent slight damage by dissolution. However, the very low incidence of apparent fragmentation of the foraminiferal tests for the CbCS indicates that the extent of dissolution was minimal.

The δ^13^C measurements were carried out in two laboratories. For *G*. *inflata*, the YT, TB, and TB2 samples were analyzed using a Finnigan-MAT253 isotope mass spectrometer coupled with a Kiel IV carbonate preparation device at the Department of Geology and Paleontology, National Museum of Nature and Science (NMNS), Tsukuba, Japan. The KG, YG, YN and YW samples were mainly analyzed using a GV Instruments IsoPrime with the Multicarb preparation system at the Center for Advanced Marine Research, Kochi University (KU), Kochi, Japan. We did not recognize any systematic differences in the results of the two laboratories for *G*. *inflata*. For *G*. *bulloides*, all samples were analyzed at the NMNS. It is known that the δ^13^C of *G*. *bulloides* varies with its size (Bemis et al. 2000; Birch et al. 2013). As we used two size fractions for *G*. *bulloides*, we found that the smaller size (YT, TB, TB2) yielded 0.27‰ lower values, on average, than the larger size (YN, YW, YG, KG) (Additional file 5). Thus, we subtracted the offset of 0.27‰ from the YN and YW samples in the composite data. International standards NBS-19 and CO-1 were used to calibrate the measured isotopic values to the Vienna Pee Dee Belemnite (VPDB) standard. The *Porites* coral standard JCp-1 was utilized as the laboratory standards. The standard deviation of the δ^13^C measurements was 0.014‰ based on 376 measurements of the NBS-19 standard at the NMNS, and 0.025‰ based on 52 measurements of the CO-1 standard at KU. The composite data for δ^13^C include the YT, TB, TB2, YW samples, and Sample YN08.

### Mg/Ca analysis

For Mg/Ca analysis, we used *G*. *bulloides* from the > 250 μm size fraction for 1.0-m and 0.2-m spaced samplings for the Yanagawa (YN), Urajiro (YW), and Kokusabata (KG) sections (Additional file 1). These foraminiferal tests had been collected previously for isotope analysis. Mg/Ca analysis was conducted on 42 samples from the CbCS (Additional files 1, 2 and 3). The temporal resolution achieved on average was 1.2 kyr, although the data are sparse for most of MIS 19 due to the low abundance of *G*. *bulloides* from this size fraction. The samples were cleaned using the “reductive” cleaning protocols of Boyle and Keigwin (1987), which includes rinsing with ultrapure water and methanol, reduction, oxidation, and leaching with 0.001M HNO_3_ in this order (Barker et al. 2003; Martin and Lea 2002). After these cleaning steps, the foraminiferal samples were dissolved in ~ 1 mL of 0.3 M HNO_3_.

Mg/Ca analysis was performed using a Finnigan ELEMENT XR sector-field inductively coupled plasma mass spectrometer at the Mutsu Institute for Oceanography, Japan Agency for Marine-Earth Science and Technology. Isotopes of three elements (^24^Mg, ^44^Ca, ^48^Ca, and ^55^Mn) were analyzed using Sc as the internal standard (Uchida et al. 2008; Kubota et al. 2010, 2019). The measurement was carried out in medium resolution mode (m/Dm = 4000). Mn/Ca was routinely monitored to evaluate the effectiveness of the removal of the Mn-oxide coating by the cleaning procedure. We used the SPEX Claritas PPT® single standard solution to make standard solutions. Four standard solutions, used to determine the concentration of each element, were prepared by successive dilutions of a multi-elemental stock standard solution (Ca, Mg, Mn) to match the concentrations of Ca (~ 0.04–5 ppm) and Mg (~ 0.08–10 ppb), and Mn (~ 0.02–2 ppb). To all samples and standard solutions, an Sc solution was added as an internal standard to adjust the Sc concentration to 1 ppb. We conducted replicate measurements of working standards (Ca ~ 1 ppm) every six samples or fewer for each run. The relative standard deviation (RSD) of Mg/Ca for 24 replicate measurements of the working standard was 1.1% for five runs, which is equivalent to ~ 0.1 °C in Mg/Ca-based temperature reconstruction. Mn/Ca values on average were 40 μmol/mol, and no values exceeded 104 μmol/mol, indicating negligible Mn-oxide contamination.

Mg/Ca values for *G*. *bulloides* were converted to a temperature scale using the calibration of Mashiotta et al. (1999) as follows:

$$ \mathrm{Mg}/\mathrm{Ca}=0.474\ \exp (0.107T) $$

where *T* represents temperature.

### Principal component analysis

#### Floral and faunal records

We conducted principal component analysis (PCA) on multi-proxy records of the CbCS, comprising floral, faunal, and geochemical data. For PCA, we selected taxa, listed in Table 1, that reflect particular water mass types, in this case the Kuroshio and Oyashio currents. For calcareous nannofossils, we used six taxa, *Calcidiscus leptoporus*, *Coccolithus pelagicus braarudii*, *Coccolithus pelagicus pelagicus*, *Umbilicosphaera* spp., *Helicosphaera* spp., and *Florisphaera profunda* (Suganuma et al. 2018; Kameo et al. 2020). The relative abundance of *F*. *profunda* was based on a count of 200 specimens of the smear slide (Suganuma et al. 2018; Kameo et al. 2020). However, the genera *Gephyrocapsa*, *Pseudoemiliania*, and *Florisphaera* account for 90% of the total calcareous nannofossils, obscuring the rare taxa that characterize typical marine environments (Suganuma et al. 2018). We therefore used the results of an additional 100 counts for subordinate taxa after 200 counts for major taxa (Kameo et al. 2020). The relative abundances of *C*. *leptoporus*, *C*. *pelagicus braarudii*, *C*. *pelagicus pelagicus*, *Umbilicosphaera* spp., and *Helicosphaera* spp. are expressed as those of the subordinate taxa, whereas *F*. *profunda* abundances are based on the total nannofossil count.

**Table 1:** The results of principal component analysis (PCA) on the Chiba composite section records. The percentage of the total variance explained and correlation coefficient (loading) for each mode are presented according to taxa

		Principal component mode					
		PC1	PC2	PC3	PC4	PC5	PC6
		Percentage of the total variance explained					
		36.3	15.4	9.7	8.2	6.5	5.1
Microfossil group	Taxa	Principal component coefficients (loadings)					
Radiolarians	*Tetrapyle* spp.	-0.14	0.14	0.34	0.15	0.49	0.12
	*Spongodiscus resurgens*	0.07	0.25	0.42	-0.05	0.16	0.33
	*Lithomelissa setosa*	0.27	-0.35	-0.07	-0.08	0.19	-0.43
	*Didymocyrtis* spp.	-0.21	0.01	-0.16	0.25	-0.36	-0.28
	*Dictyocoryne* spp.	-0.17	0.43	-0.01	0.13	-0.01	0.01
	*Cycladophora davisiana*	0.00	-0.16	-0.22	-0.34	-0.32	0.59
Calcareous nannofossils	*Calcidiscus leptoporus*	-0.20	-0.16	-0.25	0.50	0.06	0.19
	*Coccolithus pelagicus braarudii*	0.34	0.11	-0.09	-0.23	-0.10	-0.04
	*Coccolithus pelagicus pelagicus*	0.05	0.24	0.02	-0.47	0.04	-0.18
	*Umbilicosphaera* spp.	-0.35	0.10	0.08	0.00	-0.14	-0.06
	*Helicosphaera* spp.	-0.20	-0.37	0.16	0.08	0.02	0.02
	*Florisphaera profunda*	-0.33	-0.04	0.11	-0.28	0.11	-0.21
Geochemical proxies	*G. bulloides* δ^13^C	-0.26	-0.16	-0.33	-0.23	0.37	0.01
	*G. bulloides* δ^18^O	0.32	-0.08	-0.04	0.15	0.30	0.03
	*G. inflata* δ^13^C	-0.27	-0.35	0.21	-0.20	0.05	-0.09
	*G. inflata* δ^18^O	0.39	-0.15	0.14	0.20	0.00	-0.01
	*G. inflata* Mg/Ca	-0.02	0.35	-0.09	0.09	-0.05	-0.35
	δ^13^C difference (Δδ^13^C_inf-bul_)	-0.01	-0.19	0.54	0.03	-0.32	-0.09
	δ^18^O difference (Δδ^18^O_inf-bul_)	0.08	-0.07	0.18	0.04	-0.30	-0.04

For the radiolarians, we used six taxa, *Tetrapyle* spp., *Spongodiscus resurgens*, *Lithomelissa setosa*, *Didymocyrtis* spp., *Dictyocoryne* spp., and *Cycladophora davisiana*; and for the present study we present 63 horizons adding to the data published in Suganuma et al. (2018) (Additional file 3). Planktonic foraminiferal assemblage data (Suganuma et al. 2018) were not included in PCA since their temporal resolution was much lower than for calcareous nannofossils and radiolarians.

The original micropaleontological data used in this study are expressed as relative abundances (e.g., percentage of the taxon to the total fauna/flora). The sum of the relative abundance data for each sampling horizon is therefore necessarily constant (100%), violating underlying assumptions used with PCA such as the independence of each dataset (Aitchison 1982, 1986; Kucera and Malmgren 1998). Log-ratio transformation of the raw percentage data has been proposed to avoid the mathematical problems caused by this constant-sum constraint for geological data (Aitchison 1982, 2003). The effect of the constant-sum restriction on covariance and correlation matrices is removed when the relative abundances are expressed as logarithms of ratios, where the denominator is the geometric mean of the percentages in each sample (Aitchison 1982, 1986; Kucera and Malmgren 1998). In this study, the raw percentage data were transformed into log-ratio data following the method proposed by Aitchison (1982) and Kucera and Malmgren (1998). Zero elements in each data set were replaced by one-half of the lowest value of the data set (Aitchison 1982). Strictly speaking, the relative abundances of subordinate taxa for calcareous nannofossils are not equivalent to those of major taxa (e.g., *F*. *profunda*). However, the temporal variation of the subordinate taxa was comparable to that of *F*. *profunda* as ultimately standardized. The temporal resolutions of calcareous nannofossil and radiolarian records are 0.7 kyr and 0.4 kyr, respectively. Prior to PCA, the CbCS records with different time-resolutions and different variances were standardized and linearly interpolated every 100 years from 751.8 to 800.2 ka using MATLAB ® (version R2020a).

#### Geochemical records

We used the composite records of oxygen and carbon isotopes (δ^18^O and δ^13^C) for *G*. *bulloides* and *G*. *inflata*, the differences in δ^18^O and δ^13^C between the two species, and the Mg/Ca-based temperature reconstructions of *G*. *inflata* (Table 1) in PCA. Among them, the δ^13^C records are newly published in this study, and the δ^18^O and Mg/Ca data for *G*. *inflata* are published in Haneda et al. (2020a) and Suganuma et al. (2018) (Additional file 3). We excluded the Mg/Ca data for *G*. *bulloides* from PCA because the temporal resolution is low especially during MIS 19c and the following interstadials owing to the low abundance of this species within the examined interval.

We used the published Mg/Ca values of *G*. *inflata* (Suganuma et al. 2018) and converted these to temperature using the following species-specific equation for *G*. *inflata* by Anand et al. (2003):

$$ \mathrm{Mg}/\mathrm{Ca}=0.56\ \exp (0.058T) $$

where *T* denotes temperature. We chose the equation above instead of that by Anand et al. (2003) as used in Suganuma et al. (2018) because it shows realistic temperatures for the Late Holocene (Sagawa et al. 2011). Note that the process of standardization for the Mg/Ca-based temperature before PCA leads to the same PCA results as other Mg/Ca-temperature equations proposed elsewhere that give a range of absolute temperature values. The geochemical proxies presented were standardized before PCA. As with the faunal and floral data, the age was linearly interpolated every 100 years from 751.8 to 800.2 ka. PCA was performed in MATLAB® (version R2020a) using the “pca” function.

## Results

### Mg/Ca-temperature reconstruction for *G*. *bulloides*

**Fig. 2:**
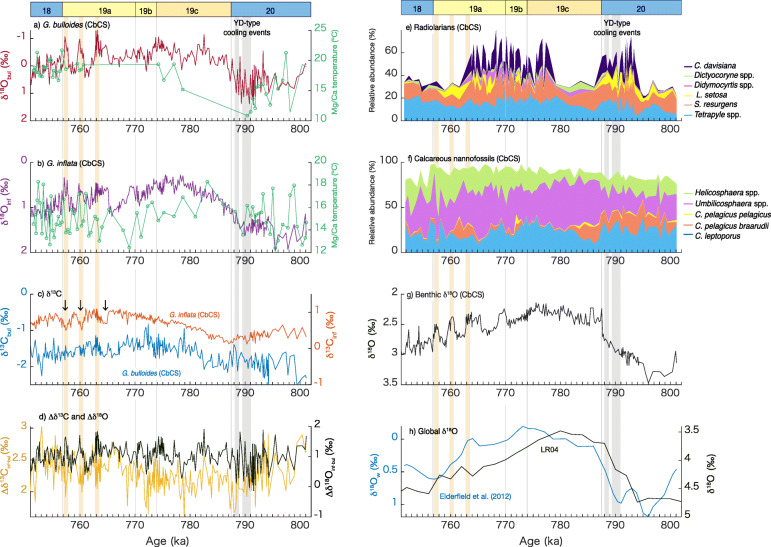
Marine records of the Chiba composite section (CbCS) showing **a** δ^18^O (red) and Mg/Ca-based winter temperature (green) for *G*. *bulloides*, **b** δ^18^O (purple) and Mg/Ca-based winter temperature (green) for *G*. *inflata* (Suganuma et al. 2018; Haneda et al. 2020a), **c** δ^13^C for *G*. *bulloides* (blue) and for *G*. *inflata* (orange), **d** Δδ^13^C_inf-bul_ (yellow) and Δδ^18^O_inf-bul_ (black), **e**, **f** microfossil relative abundances for **e** radiolarians (Suganuma et al. 2018 and this study), **f** calcareous nannofossils (Kameo et al. 2020), **g** benthic δ^18^O in the CbCS (Suganuma et al. 2018), **h** comparison to global mean δ_18_O of water (δ_18_O_w_) of Elderfield et al. (2012) (blue) and the benthic δ^18^O stack of Lisiecki and Raymo (2005) (black). Younger Dryas-type cooling events during Termination IX and distinct millennial events in δ^18^O of *G*. *bulloides* during MIS 19a are highlighted in gray and yellow. Arrows in Fig. 3c represent negative peaks in δ^13^C of *G*. *inflata* mentioned in the text. Figure 2 illustrates the data from 801 to 751 ka.

The Mg/Ca values for *G*. *bulloides* range between 1.51 and 4.62 mmol/mol: 10.8–21.8 °C on the temperature scale (Fig. 2a and Additional file 3). The Mg/Ca temperature shows relatively high values (~ 19 °C) at ~800 ka, decreasing to the lowest values (~ 11 °C) during termination IX. Subsequently, the temperature increased to ~ 19 °C in late MIS 19c and maintained high values until MIS 18. As mentioned above, the temporal resolution is low for MIS 19.

### Carbon isotopes

The average and standard deviation of the composite δ^13^C for *G*. *inflata* (δ^13^C_inf_) over the entire succession from 801.13 to 747.56 ka (*n* = 336) is 0.65‰ and 0.26‰, respectively. δ^13^C_inf_ varies between − 0.15 and 1.11‰ (Additional file 3), showing low values from MIS 20 to early MIS 19c and high values in MIS 19b through early MIS 18, and a decreasing trend toward the middle of MIS 18 (Fig. 2c). Three distinctive negative peaks were found in late MIS 19a with an amplitude of 0.3–0.4‰.

The average and standard deviation of the composite δ^13^C for *G. bulloides* (δ^13^C_bul_) over the entire succession from 801.13 to 747.56 ka (*n* = 309) is − 1.63‰ and 0.28‰, respectively. δ^13^C_bul_ values are lower than for δ^13^C_inf_, varying between − 2.55 and − 0.82‰ (Additional file 3). The temporal trend in δ^13^C_bul_ is different from that in δ^13^C_inf_, showing low values in MIS 20 through early MIS 19c (Fig. 2c). δ^13^C_bul_ increases during MIS 19c, reaches highest values during MIS 19b, and decreases thereafter. Short-period oscillations are superimposed on these long-term variations. Two negative δ^13^C_bul_ peaks at 764.5 ka and 760.0 ka in MIS 19a correlate with two of three distinctive negative δ^13^C_inf_ peaks, but the following δ^13^C_inf_ peak seem not to be associated with the δ^13^C_bul_ record.

### PCA results

**Fig. 3:**
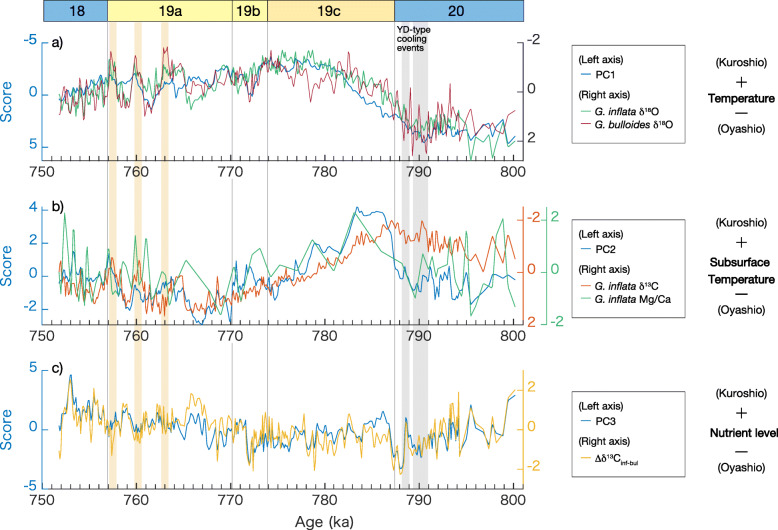
Results of principal component analysis (PCA) compared to related geochemical proxies in the Chiba composite section, for **a** leading mode (PC1), **b** second mode (PC2), and **c** third mode (PC3). The geochemical proxies presented are standardized. Highlighted events are the same as for Fig. 2

The PCA results indicate that the leading mode (PC1) carries 36.3% of the total variance, characterized by high loadings in the δ^18^O records for *G*. *inflata* (δ^18^O_inf_) and *G*. *bulloides* (δ^18^O_bul_), the calcareous nannofossil *C*. *pelagicus braarudii*, and the radiolarian *L*. *setosa* (Table 1). Temporal variation in PC1, the best representative of the δ^18^O data, exhibits a glacial–interglacial cycle, showing high scores in MIS 20 and a decreasing trend at Termination IX (Fig. 3a). PC1 shows lowest scores in middle to late MIS 19c and increases subsequently in association with short-period variations. Although millennial-scale fluctuations superimposed on orbital-scale variability are also prominent in the planktonic δ^18^O_bul_ and δ^18^O_inf_ records during Termination IX, MIS 19b, and MIS 19a (Suganuma et al. 2018; Haneda et al. 2020a), these fluctuations are not fully expressed in PC1 (Fig. 3a). In particular, three negative peaks are prominent in both the δ^18^O_bul_ and δ^18^O_inf_ records during MIS 19a (763–757 ka) but they are marginally seen in PC1. In contrast, a prominent positive peak in PC1 at ~772 ka in MIS 19b coincides with the positive peak in δ^18^O_bul_. A positive peak in PC1 at ~761 ka in MIS 19a is also prominent and correlated to δ^18^O_bul_ and δ^18^O_inf_. PC1 also shows negative loadings in the calcareous nannofossil taxa *Umbilicosphaera* spp. and *F*. *profunda* and in δ^13^C_inf_ and δ^13^C_bul_, in descending order (Table 1).

The second mode (PC2) carries 15.4% of the total variance and is characterized by high loadings in the Mg/Ca-temperature for *G*. *inflata* and the radiolarian *Dictyocoryne* spp. (Table 1). Negative loadings are found in δ^13^C_inf_, the calcareous nannofossil *Helicosphaera* spp., and radiolarian *L. setosa*. PC2 exhibits mostly negative scores in MIS 20, reaches highest scores in early MIS 19c, and shows a decreasing trend in MIS 19c through MIS 19a (Fig. 3b). This rapid transition following the earlier peak in MIS 19c contrasts with the more gradual changes seen in PC1. The Mg/Ca-temperature of *G*. *inflata* and the δ^13^C_inf_ record oscillate greatly during late MIS 19a and in MIS 18, but the millennial-scale variations of PC2 exhibit more similarity to δ^13^C_inf_ than to the Mg/Ca-temperature of *G. inflata*.

The third mode (PC3) contributes 9.7% of the total variance and is dominated by variations in the radiolarian *S*. *resurgens, Tetrapyle* spp., and Δδ^13^C_inf-bul_ (Table 1). The calcareous nannofossil *C. leptoporus*, radiolarian *C. davisiana*, and δ^13^C_bul_ all show negative loadings for PC3. PC3 does not display a typical glacial–interglacial pattern but oscillates greatly on the millennial to multi-millennial scale over the entire examined interval (Fig. 3c).

## Discussion

### Interpretation of geochemical proxies

The planktonic foraminifer *G*. *bulloides* is most abundant at depths shallower than 100 m in the Pacific Ocean off the Japanese archipelago (Arikawa 1983; Kuroyanagi and Kawahata 2004; Oba and Hattori 1992). This species tends to be abundant in winter in the Okinawa Trough (Yamasaki and Oda 2003; Yamasaki et al. 2010; Xu et al. 2005) and Ryukyu Trench (Xu et al. 2005), but its flux depends on the position of the Kuroshio Current in the Okinawa Trough (Xu et al. 2005). In contrast, large fluxes of *G*. *bulloides* have been observed in spring (April–May) and autumn (October–November) in the subarctic region (Itou 2000; Kuroyanagi et al. 2002) and Kuroshio–Oyashio mixed zone (Oda and Yamasaki 2005). Nevertheless, the flux of *G*. *bulloides* depends greatly on the meanders of the Kuroshio Current and associated nutrient supply in the Kuroshio–Oyashio mixed zone (Oda and Yamasaki 2005). Thus, Oda and Yamasaki (2005) concluded that *G*. *bulloides* attains its maximum abundance at times of greatest food availability. Although the highest flux of *G*. *bulloides* would be anticipated during the phytoplankton blooming season, the reconstructed Mg/Ca-SST of *G*. *bulloides* for the late Holocene was ~ 3 °C lower than the alkenone-based SST that represents a blooming season in early summer (Sagawa et al. 2006; Yamamoto et al. 2005). Given that *G*. *bulloides* is a surface-dwelling species, its calcification depth is likely similar to the phototrophic haptophyte algae that produce the alkenones. We therefore interpret *G*. *bulloides* abundances as reflecting the early months of the blooming season (i.e., late winter through spring) in the Kuroshio–Oyashio mixed zone.

The planktonic foraminifer *G*. *inflata* is a subsurface dweller and typifies the central water mass near the northern margin of the Kuroshio Current (Coulbourn et al. 1980; Vincent and Berger 1981). In the western North Pacific, its calcification depth toward the south is deep (~ 300–400 m at 28° N; Sagawa et al. 2011) while slightly shallower (> 100 m) in the Kuroshio–Oyashio mixed zone at 35° N (Oba and Hattori 1992). *Globorotalia inflata* prefers dwelling in a constant temperature at subsurface depths (100–400 m) during the cold season in the Kuroshio–Oyashio mixed zone (Oda and Yamasaki 2005). We therefore interpret the geochemical proxies of *G*. *inflata* to reflect the subsurface environment with a bias toward the winter season (Suganuma et al. 2018).

The foraminiferal δ^18^O is primarily determined by temperature and the δ^18^O of the ambient water (e.g., local salinity and global ice volume) with small species-specific disequilibrium factors (< 1‰, Rohling and Cooke 1999). Suganuma et al. (2018) posited for the CbCS that a difference in δ^18^O between *G*. *inflata* and *G*. *bulloides* (δ^18^O_inf_ – δ^18^O_bul_ = Δδ^18^O_inf-bul_) reflected a vertical density (representing dominantly temperature) gradient between subsurface and surface waters and that a large Δδ^18^O_inf-bul_ reflected stratification of the ocean.

The foraminiferal δ^13^C is also determined by equilibrium fractionation and disequilibrium factors, but its interpretation is complicated by a highly variable ambient seawater δ^13^C and disequilibrium factors that are not well known (Rohling and Cooke 1999). Off east-central Japan today, the δ^13^C of the dissolved inorganic carbon (DIC, hereafter δ^13^C_DIC_) in the ocean is primarily determined by fluctuations between Kuroshio and Oyashio waters (Oba et al. 2006) that control nutrients at the surface, local primary productivity, and export production (Rohling and Cooke 1999). Since the δ^13^C_DIC_ gradient in the water column between the surface and subsurface is steep, changes in the depth of calcification may cause deviations in the quantity of δ^13^C observed in foraminiferal calcite at the studied site (Oba et al. 2006). At the surface, the δ^13^C_DIC_ is higher in the Oyashio Current region (~ 2‰) than in the Kuroshio Current region (~ 1‰) (Oba et al. 2006). This surface δ^13^C_DIC_ gradient is caused by high primary productivity in the Oyashio Current, where phytoplankton preferentially utilize ^12^C during photosynthesis. Generally, δ^13^C_DIC_ has high values at the surface that diminish with depth owing to the remineralization of organic matter in the water column. In the Oyashio region, the δ^13^C_DIC_ for the subsurface (100–400 m) rapidly decreases from ~ 0 to ~− 0.7‰ with depth, while in the Kuroshio region it decreases from ~ 0.8 to ~ 0.5‰ (Oba et al. 2006).

The δ^13^C disequilibrium is species (or specimen) specific and related to the (1) photosynthetic activity of symbionts, (2) incorporation of metabolic CO_2_, (3) growth rate, and (4) carbonate chemistry in the ambient waters (Rohling and Cooke 1999). The non-spinose species are less affected by these biological fractionation effects because of their lack of symbionts (Ravelo and Fairbanks 1995). Ravelo and Fairbanks (1995) suggested that disequilibrium fractionations (kinetic and biological fractionation effects) vary as a function of temperature based on a positive δ^13^C–δ^18^O relationship in *G*. *inflata* from core-top samples in the tropical Atlantic. *Globigerina bulloides* is a spinose but non-symbiont-bearing species and has the potential to record in situ δ^13^C_DIC_. The δ^13^C_bul_ varies as a function of temperature; decreasing 0.11‰/°C as more δ^13^C-depleted respired CO_2_ is incorporated into shell carbon at a higher metabolic rate (Bemis et al. 2000). This metabolic-related temperature effect is mainly seen in opportunistic species, such as *G*. *bulloides*, which have short life cycles and likely have high metabolic rates throughout life (Birch et al. 2013). The δ^13^C_bul_ in the CbCS shows lower values than that of *G*. *inflata*, which is consistent with the previous study by Oba et al. (2006).

At the studied site, the low SST of the Oyashio Current is expected to have increased the δ^13^C_bul_ because of the temperature effect on the foraminiferal calcite δ^13^C (Bemis et al. 2000). On the other hand, concerning the change in the surface δ^13^C_DIC_ between the Kuroshio and Oyashio water regions, the influence of the Oyashio is expected to have increased the δ^13^C_bul_ (Oba et al. 2006). Indeed, the δ^13^C_bul_ off east-central Japan exhibited higher values during the Last Glacial Maximum when the Oyashio influence became greater (Oba et al. 2006). In contrast, the δ^13^C_DIC_ gradient between the Kuroshio and Oyashio water regions in the subsurface is opposite to that of the surface: the δ^13^C_DIC_ of the Oyashio waters is lower than the Kuroshio (Oba et al. 2006). However, the low temperature in the Oyashio waters would increase the δ^13^C_inf_ (Ravelo and Fairbanks 1995), and therefore the temperature effect acts to diminish the latitudinal δ^13^C_DIC_ gradient in the Kuroshio–Oyashio mixed zone.

The vertical gradient in foraminiferal δ^13^C (δ^13^C of *G*. *inflata* – δ^13^C of *G*. *bulloides* = Δδ^13^C_inf-bul_), as proposed by Oba et al. (2006), is another measure of whether the Kuroshio or Oyashio waters were dominant in terms of nutrient levels and productivity. As foraminiferal δ^13^C includes a global δ^13^C_DIC_ change for the entire ocean during glacial–interglacial cycles, the subtracting process proposed by Oba et al. (2006) excludes the global signal, and therefore the Δδ^13^C_inf-bul_ indicates local δ^13^C_DIC_ change. The vertical gradient in δ^13^C_DIC_ between subsurface and surface waters is greater in the Oyashio waters than the Kuroshio waters (Oba et al. 2006). Given that there is a ~ 2‰ offset between the surface δ^13^C_DIC_ and δ^13^C_bul_, the foraminiferal Δδ^13^C_inf-bul_ would be smaller in the Oyashio waters and larger in the Kuroshio waters (Oba et al. 2006). Indeed, the Δδ^13^C_inf-bul_ was smaller during glacials (MIS 2 and MIS 6) and larger during interglacials (MIS 1 and MIS 5) in the Kuroshio-Oyashio mixed zone (Oba et al. 2006).

### Multi-proxy approach for the Chiba composite section

The published high-resolution geochemical and relatively high-resolution faunal and floral records (Suganuma et al. 2018), in addition to planktonic δ^13^C data presented in this study, provide an excellent opportunity to examine inter-proxy relationships and enhance our understanding of the paleoceanography based on robust evidence from multiple proxies. Suganuma et al. (2018) overviewed temporal variations in the foraminiferal δ^18^O records at the CbCS; both benthic and planktonic δ^18^O values show pronounced glacial–interglacial cycles from MIS 20 to 18 (Fig. 2a, b, and g). Particularly, Suganuma et al. (2018) mentioned that the warmer SSTs during the later part of MIS 19, as inferred from the low δ^18^O_bul_, most likely correspond to the northward shift of the Kuroshio–Oyashio boundary, supported by stratified conditions based on the increased *F*. *profunda* and Δδ^18^O_inf-bul_.

**Fig. 4:**
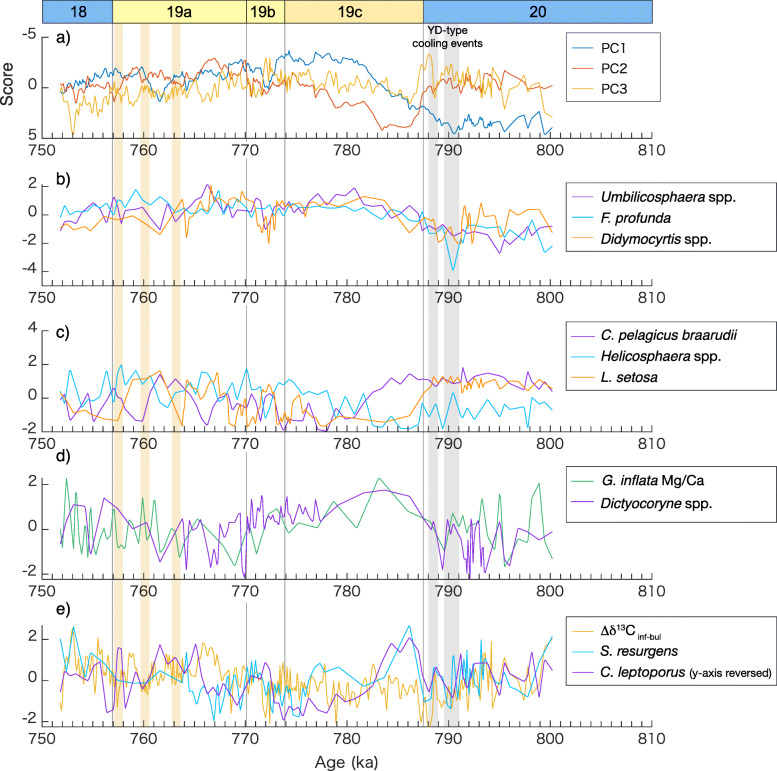
Results of principal component analysis (PCA) compared to microfossil relative abundances and geochemical proxies. **a** Scores for PC1, PC2, and PC3. **b**, **c** Standardized microfossil relative abundances for PC1, **d** for PC2, and **e** for PC3, but the *y* axis for *C*. *leptoporus* is reversed. Highlighted events are the same as Fig. 2

The temporal variation in PC1 resembles variation shown by the δ^18^O (positive loading) and δ^13^C (negative loading) among the geochemical proxies and tends to display negative scores from late MIS 19c to MIS 18 (Fig. 3a). The calcareous nannofossils *F*. *profunda* and *Umbilicosphaera* spp., negatively correlated to the score for PC1, are the Kuroshio- (or tropical water) related taxa (Table 1 and Fig. 4). *Umbilicosphaera* spp. are abundant today in the Kuroshio axis, and *F*. *profunda* is abundant in warm waters of the central Pacific (Tanaka 1991). The radiolarian taxon *Didymocyrtis* spp., which correlates negatively but weakly with PC1 (Table 1, Fig. 4a and b), is also the typical dominant taxon in the warm shallow waters of the North Equatorial Current (Matsuzaki and Itaki 2017). The temporal variations in *Didymocyrtis* spp. agree well with those of the calcareous nannofossils *F*. *profunda* and *Umbilicosphaera* spp. (Fig. 4b). The radiolarians *Tetrapyle* spp. and *Dictyocoryne* spp., which are subtropical taxa characteristic of the Kuroshio Current region (Matsuzaki and Itaki 2017), exhibit negative loadings, although these are relatively weak.

The calcareous nannofossil *C*. *pelagicus* is found in the Kuroshio–Oyashio mixed zone off the northeastern coast of the Japanese archipelago, and is especially abundant in the northern part (Tanaka 1991). *Coccolithus pelagicus* in Tanaka (1991) may include *C*. *pelagicus braarudii* and *C*. *pelagicus pelagicus*, whereas we separate *C*. *pelagicus braarudii* from *C*. *pelagicus pelagicus* (Kameo et al. 2020). Although these two subspecies have their ecological preferences in the subpolar Atlantic Ocean (i.e., *C*. *pelagicus pelagicus* prefers colder waters than *C*. *pelagicus braarudii*) (Narciso et al. 2006), we treat them together as a typical representative of cooler surface waters in this study based on the observation by Tanaka (1991). Thus, we interpret *C*. *pelagicus braarudii* as a representative of the Oyashio or mixed waters at the CbCS. The PCA result indicates that *C*. *pelagicus braarudii* and the radiolarian *L*. *setosa* show positive loadings for PC1 (Table 1, Fig. 4c). The radiolarian *L*. *setosa* is a subarctic to arctic coastal species, and absent offshore (Matsuzaki and Itaki 2017). These consistent faunal and floral signatures indicate that PC1 is related to the shallow waters of the Kuroshio or Oyashio/mixed waters. Therefore, together with the planktonic δ^18^O, we interpret PC1 as an indicator of the relative contributions of the Kuroshio and Oyashio/mixed waters; the negative scores of PC1 representing the dominance of the shallow Kuroshio waters and vice versa.

PC2 nearly parallels the Mg/Ca of *G*. *inflata* and shows highest values during early MIS 19c (Fig. 3b). The subsurface temperature signal, likely coupled with the variation in the subsurface warm water mass (NPTW) probably transported by the Kuroshio Current, is therefore closely linked to PC2. The negative loading in δ^13^C_inf_ is also high. The negative correlation between Mg/Ca-temperature and δ^13^C_inf_ suggests that the temperature effect is not negligible on the δ^13^C_inf_. Among the radiolarian taxa, a high loading for PC2 is found in *Dictyocoryne* spp. (Table 1). The temporal variation in *Dictyocoryne* spp. has an obvious similarity to that in the subsurface temperature (Fig. 4d). Among the genus *Dictyocoryne*, *D*. *profunda* and *D*. *truncatum* are widely found in the equatorial Pacific and the Kuroshio region of the western North Pacific, but have greatest abundances in the equatorial region (Matsuzaki et al. 2016, 2020; Matsuzaki and Itaki 2017). *Dictyocoryne muelleri* is also abundant in the surface Kuroshio water but found even at the subsurface (100–200 m) and intermediate (500–700 m) depths in the East China Sea (Matsuzaki et al. 2016). An increase in *Dictyocoryne* spp. at the CbCS, therefore, suggests the more significant transport by the Kuroshio Current from the equatorial/subtropical region. Given that the subsurface temperature contributes significantly to PC2, we further suggest that *Dictyocoryne* spp. is associated with the subsurface Kuroshio water, which is consistent with the depth habitat of *D*. *muelleri*.

In contrast, the loading for δ^18^O_bul_ is low in PC2, suggesting that the surface temperature does not contribute to PC2; PC2 is uniquely linked to the subsurface temperature. Here, *Helicosphaera* spp. mainly consists of *Helicospharea carteri*, *Helicosphaera hyalina*, and *Helicosphaera pavimentum* with rare *Helicosphaera wallichii* (Kameo et al. 2020). This taxon is abundant in the East China Sea and also found in coastal areas along the southern part (< 36° N) of the Japanese archipelago (Tanaka 1991). The coastal taxa (*L*. *setosa* and *Helicosphaera* spp.) exhibit negative loadings for PC2, reflecting the characteristics of the coastal waters (cool and less saline) in contrast to the subsurface water of the Kuroshio Current (warm and saline). The decreasing trend in PC2 during middle and late MIS 19c coincides with a lowering of the subsurface temperature, suggesting less influence of NPTW. This oceanic change may be favorable to the coastal taxa as the relative abundances of *Helicosphaera* spp. and *L*. *setosa* begins to increase during middle and late MIS 19c.

The temporal variation in PC3 lacks a typical glacial–interglacial pattern and is correlated with Δδ^13^C_inf-bul_. According to the interpretation of Oba et al. (2006), a high Δδ^13^C_inf-bul_ is led by a stratified ocean that is closely associated with the more significant influence of the Kuroshio Current. Our results indicate that a high Δδ^13^C_inf-bul_ mostly corresponds to a high Δδ^18^O_inf-bul_ (Fig. 2d), being consistent with the nutrient levels inferred by Δδ^18^O_inf-bul_. The high Δδ^18^O_inf-bul_ is interpreted as a stratified water column and vice versa (Suganuma et al. 2018). Concerning the faunal data, PC3 indicates a relatively high correlation to the radiolarian *S*. *resurgens.* High abundances of this species are typically characteristic of the mixture of subtropical and subarctic waters carried by the Kuroshio and Oyashio currents where they converge and suggest nutrient-rich waters (Matsuzaki and Itaki 2017). However, the temporal variation of *S*. *resurgens* is in opposition to the nutrient levels suggested by the Δδ^13^C_inf-bul_ record. Although *S. resurgens* is a transitional species abundant in a broad temperature zone between 16 and 23 °C (Matsuzaki and Itaki 2017), we are inclined to the interpretation that this species is better adapted to the oligotrophic waters of the Kuroshio Current than the nutrient-rich Oyashio waters in the Kuroshio-Oyashio mixed zone. On the other hand, the calcareous nannofossil *C*. *leptoporus* favors offshore waters far from the coast of the Japanese archipelago, as with *F*. *profunda*, but is more abundant toward the north (26–38° N) (Tanaka 1991). The temporal variation of this species is opposite to those of Δδ^13^C_inf-bul_ and *S*. *resurgens* (Fig. 4e), suggesting that *C*. *leptoporus* is more abundant in nutrient-rich (low Δδ^13^C_inf-bul_) waters. The contrast between *C*. *leptoporus* and *S*. *resurgens* and their relationship to the Δδ^13^C_inf-bul_ record appears contradictory, but implies an inverse water mass preference in terms of nutrient status. However, we note that the temperature factor cannot be negligible in the CbCS, as mentioned above for δ^13^C_inf_, suggesting that the Δδ^13^C_inf-bul_ signal is a mixture of nutrient levels and the vertical temperature gradient.

A Younger Dryas-type cooling event during Termination IX, reflected in the δ^18^O_bul_ record as pronounced positive peaks at around 788 ka and 791 ka (Suganuma et al. 2018; Haneda et al. 2020a), is well expressed in PC3 (Fig. 3c). A rapid increase in PC3 at ~787 ka coincides with PC2 and shows a distinct positive peak early in MIS 19c (Fig. 3b). Similar rises are seen in several stratification indices (Δδ^18^O_bull-benthic_, Haneda et al. 2020a; Δδ^18^O_inf-bul_ and Δδ^13^C_inf-bul_, this study; Fig. 2d), suggesting stratification and probably reduced nutrient levels after the second Younger Dryas-type cooling event. These changes indicate a rapid reorganization of the water column structure along with subsurface warming approaching the interglacial climate of MIS 19. Given that the relative abundances of *Dictyocoryne* spp. and *S*. *resurgens* mark rapid rises respectively (Fig. 4d and e), these taxa could have responded swiftly to the significant oceanographic changes following the Younger Dryas-type cooling event.

PC3 exhibits millennial-scale variability across the entire interval of study, with short-period variations mostly positively correlated to both Δδ^13^C_inf-bul_ and Δδ^18^O_inf-bul_ except for a 765–752 ka interval from MIS 19a to MIS 18 (Fig. 2 d and Fig. 3c). During the exceptional interval (765–752 ka) mentioned above, a high Δδ^13^C_inf-bul_ is correlated to a low Δδ^18^O_inf-bul_, which is not merely explained by a Kuroshio-Oyashio oscillation. In particular, three prominent negative δ^18^O_bul_ peaks, at 763 ka, 760 ka, and 757 ka, which also coincide with the low Δδ^13^C_inf-bul_, are apparent as negative scores in PC3. These opposing Δδ^13^C–Δδ^18^O relationships suggest a complicated mechanism underlying the Δδ^13^C_inf-bul_ record.

In summary, the two modes, PC1 and PC2, generally follow glacial–interglacial cyclicity on an orbital scale, confirming that the global climate rhythm dominantly controls half of the total variance (PC1 + PC2 = 51.7%) of the multi-proxy records in the CbCS. The relationships between the relative abundances of individual taxa and water mass characteristics are generally consistent among the microfossil groups and geochemical proxies (Figs. 3 and 4).

### Comparison among east Asian regional records and climatological interpretation

The East Asian monsoon system is generated by seasonal thermal contrasts between the Eurasian continent and the Indo-Pacific Ocean, producing seasonal reversals in the prevailing wind and precipitation that result in wet summers and dry winters (Wang 2006). Humidity in East Asia is therefore mainly controlled by the East Asian summer monsoon (EASM) system whose variation can be recorded in Asian terrestrial proxies such as the magnetic susceptibility record of the Chinese Loess Plateau (Guo et al. 2009). Meanwhile, the EAWM, generated during winter by contrasts in atmospheric pressure between the Siberian High on the Eurasian continent and AL in the North Pacific, plays a crucial role in determining the winter wind strength and associated North Pacific gyres. These seasonal differences operate on an orbital scale where they are clearly archived in the Chinese loess plateau during MIS 20–18 (Hao et al. 2012).

**Fig. 5:**
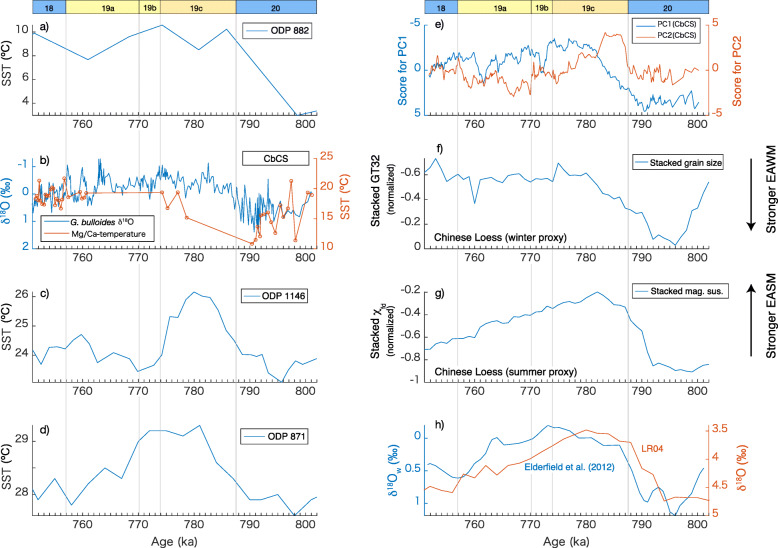
Western Pacific sea-surface temperature (SST) records for **a** subarctic ODP Site 882 based on alkenones (Martínez-García et al. 2010), **b** the Chiba Composite Section (CbCS) based on *G*. *bulloides* δ^18^O (Suganuma et al. 2018; Haneda et al. 2020a) and Mg/Ca-based temperature (this study), **c** ODP Site 1146 in the South China Sea based on alkenones (Herbert et al. 2010), and **d** tropical ODP Site 871 based on Mg/Ca (Dyez and Ravelo 2014); in comparison with **e** principal component scores for the CbCS, **f**, **g** proxies from the Yimaguan and Luochuan sections of the Chinese Loess Plateau showing **f** stacked grain size and **g** stacked magnetic susceptibility records (Hao et al. 2012), **h** benthic δ^18^O_w_ of Elderfield et al. (2012) (blue) and benthic δ^18^O stack of Lisiecki and Raymo (2005) (orange)

The late Quaternary magnetic susceptibility records, the proxy for the EASM, in the Chinese Loess Plateau are synchronous with the global benthic δ^18^O stack (Hao et al. 2012) (Fig. 5g and h). In contrast, the grain-size records for Chinese loess, the proxy for the EAWM, generally lack the “saw-tooth” shape typical of the marine δ^18^O curve during MIS 19–18 (Fig. 5f and h), implying that global climate (i.e., global ice volume, atmospheric CO_2_ concentration) has not been a dominant controlling factor on the EAWM (Hao et al. 2012). In particular, middle MIS 19c through MIS 18 are characterized by a pronounced weak EAWM (Hao et al. 2012). These previous studies proposed that this weak EAWM, probably associated with a weak Siberian High, would be caused by the moderate summer insolation minima (Hao et al. 2012) or the enhanced winter insolation in the Northern Hemisphere (Suganuma et al. 2018).

As discussed above, PC1 is a measure of the influence of the Kuroshio Current at the CbCS. We further interpret variations in PC1 to be coupled with the latitude of the boundary between the subtropical and subarctic gyres in the North Pacific (Yamamoto et al. 2005; Suganuma et al. 2018) because surface taxa of the Kuroshio and Oyashio/mixed water and the planktonic δ^18^O dominantly contribute to PC1. The temporal variation pattern in PC1 is more similar to the EAWM proxy than the EASM proxy in terms of the gradual transition during the termination and the long duration of the warmest interglacial conditions (Fig. 5e–g). These similarities between PC1 and the EAWM proxy suggest that the EAWM exerts influence on many of the marine proxies of the CbCS. Because the wind-stress curl, which is the primary driver of the North Pacific gyres, is greater during winter than summer, it is reasonable to consider that wintertime climate signals are actively recorded in the discussed marine proxies of the CbCS. However, PC1 shows a long-term increasing trend after the peak of MIS 19c. This trend is similar to global ice volume and the EASM proxy, suggesting that PC1 is subject to the global signal and EASM as well as EAWM in the earlier in MIS 19. Although the mostly negative scores for PC1 infer a northerly position for the Kuroshio–Oyashio boundary during late MIS 19c through early MIS 18, millennial-scale variations are superimposed on the long-term trend. MIS 19b and 19a are especially represented by distinct millennial-scale oscillations. Apparent positive peaks in PC1 at ~772 ka and ~761 ka correlate with positive peaks in planktonic δ^18^O and to decreases in the relative abundances of *Umbilicosphaera* spp. and *Didymocyrtis* spp., and to increases in *L. setosa* (Fig. 4), indicating that the millennial-scale oscillations in PC1 during MIS 19b and 19a are closely linked to Kuroshio–Oyashio variations. Thus, the Kuroshio–Oyashio boundary likely shifts southwards when the scores for PC1 shift to positive values (stadials), whereas the Kuroshio water dominates and the gyre boundary shifts northwards when the scores for PC1 become negative (interstadials). The stadial at ~772 ka in MIS 19b and at ~761 ka in MIS 19a are predominant in the CbCS records. In contrast to the high-resolution records from the CbCS, however, millennial-scale variations are not clearly observable in the Chinese monsoon proxies probably because of their relatively low temporal resolution.

As PC2 represents the subsurface, as discussed above, it further helps to understand the water column structure in the Kuroshio–Oyashio mixed zone. The temporal pattern in PC2 begins to deviate from PC1 from early MIS 19c (Fig. 5e), suggesting a change in subsurface water that is independent of the ocean surface. Today, increases in the salinity of NPTW, the subsurface water mass in the Kuroshio Current, are associated with the intensification of the AL and result in an accelerated subtropical gyre (Suga et al. 2000). As the Kuroshio Current carries NPTW into the mid-latitudes, the subsurface temperature in the Kuroshio–Oyashio mixed region is presumably affected by the intensity of the subtropical gyre; relaxation of the subtropical gyre may be associated with subsurface cooling and freshening and vice versa. The reduced gyre circulation may occur independently of the north–south shift of the gyre boundary (PC1), as suggested by modern observations (Sugimoto and Hanawa 2009), potentially explaining the difference in the temporal variations between PC1 and PC2. Thus, we infer that the northerly shift in the gyre boundary during late MIS 19c and early MIS 19a after the stadial at ~772 ka was not associated with subsurface warming due to the suppressed gyre circulation itself caused by the weak AL. This interpretation is consistent with a decrease in the tropical *Dictyocoryne* spp. that would be transported by the Kuroshio Current: this taxon is therefore a good indicator of the gyre circulation. In contrast, the millennial-scale variations in PC2 tend to coincide with changes in PC1 during late MIS 19a, suggesting a mechanism different from the previous period. 

Furthermore, an increase in the production and advection of NPIW might have contributed to subsurface cooling during mid-MIS 19c. As was suggested by Itaki and Ikehara (2004), the production of the Okhotsk Sea Intermediate Water that contributes to NPIW is coupled tightly with winter warmth and significantly increased during a warm interval of the Holocene. We infer that moderate winters from mid-MIS 19c led to an increase in NPIW advection into the subsurface layer of the Kuroshio–Oyashio mixed zone.

### Northwestern Pacific during MIS 20–18 and comparison to MIS 2–1

**Fig. 6:**
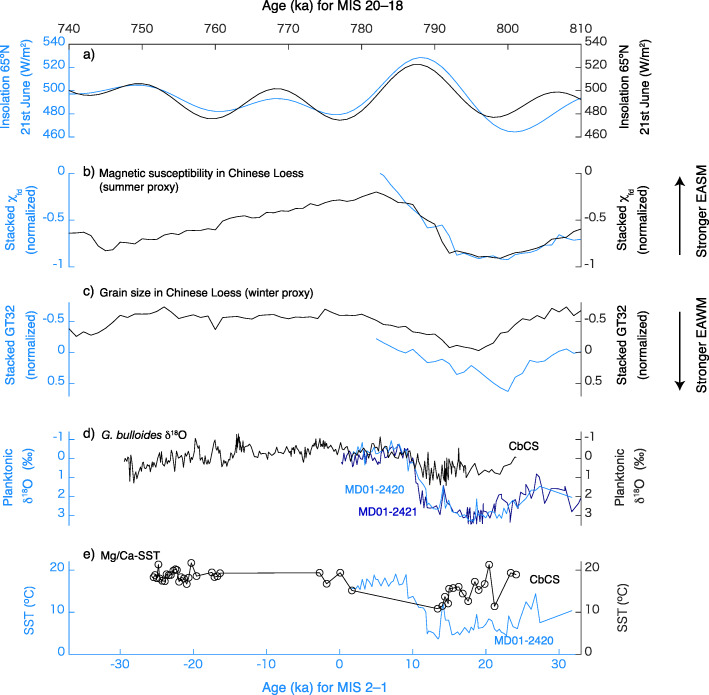
A comparison between MIS 2–1 and MIS 20–18 with bottom axis for the age for MIS 2–1 and top axis for the age for MIS 20–18. **a** Northern Hemisphere summer insolation at 65° N for MIS 2–1 (blue) and MIS 20–18 (black), **b**, **c** proxies from the Yimaguan and Luochuan sections of the Chinese Loess Plateau showing **b** stacked grain size (EAWM) and **c** stacked magnetic susceptibility (EASM) (Hao et al. 2012) for MIS 2–1 (blue) and MIS 20–18 (black), **d**, **e** the planktonic foraminiferal (*G. bulloides*), **d** δ^18^O, and **e** Mg/Ca-based SST in the Kuroshio–Oyashio mixed zone for MIS 2–1 in MD01-2420 (blue, from Sagawa et al. 2006) and MD01-2421 for δ^18^O only (dark blue, from Oba et al. 2006), and MIS 20–18 (black, from the CbCS)

To capture the regional temperature structure in the western North Pacific during MIS 20–18 and its relation to the global and Asian regional climate signals, we compare the published SST records between 5° N and 50° N to the newly reported Mg/Ca-SST (*G*. *bulloides*) record for the CbCS, although the data treated here do not contain the glacial maximum for MIS 18 (Table 2; Fig. 5 a–d and Fig. 6). SST records for MIS 20–18 have been reported for the WPWP (Mg/Ca of *Globigerinoides ruber* from Ocean Drilling Program [ODP] Site 871; Dyez and Ravelo 2014), South China Sea (alkenones from ODP Site 1146; Herbert et al. 2010), and the subarctic (alkenones from ODP Site 882; Martínez-García et al. 2010) (Figs. 5a, c, and d). The benthic δ^18^O and magnetic susceptibility data used for their age models are illustrated in Additional file 6 for comparison. The benthic δ^18^O stratigraphy combined with magnetostratigraphy enable us to compare the SST records within an uncertainty of ca. 5 ka (Suganuma et al. 2021).

**Table 2:** Western Pacific SST records compared in this study for MIS 2–1 and MIS 20–18 (Fig. 7). *Ages for inception of MIS 20 and termination of MIS 18 are based on the oldest and youngest ages of our data

Site	Longitude (ºE)	Latitude (ºN)	Proxy	Mg/Ca Calibration	References	Average SST (ºC)				Standard deviation of SST (ºC)				Number of data for averaging			
						MIS 18 (747.0*–756.9 ka)	MIS 19a, 19b (756.9–773.9 ka)	MIS 19c (773.9–787.5ka)	MIS 20 (787.5–801.0*ka)	MIS 18	MIS 19a, 19b	MIS 19c	MIS 20	MIS18	MIS19a, 19b	MIS19c	MIS 20
ODP 871	172.35	5.55	Mg/Ca (*Globigerinoides ruber*)	Dyez and Ravelo (2013)	Dyez and Ravelo (2014)	28.1	28.6	29.1	27.9	0.3	0.4	0.3	0.2	4	6	5	5
ODP 1146	116.27	19.46	Alkenone		Herbert et al. (2010)	24.1	24.1	25.4	23.7	0.3	0.4	0.4	0.3	7	8	10	11
CbCS	140.13	35.28	Mg/Ca (*Globigerina bulloides*)	Mashiotta et al. (1999)	This study	18.4	18.8	17.7	15.2	1.0	0.5	2.1	3.1	17	5	4	16
ODP 882	167.58	50.35	Alkenone		Martínez-García et al. (2010)	8.8	9.6	9.8	3.0			1.1		1	1	3	1
Site	Longitude (ºE)	Latitude (ºN)	Proxy	Mg/Ca Calibration	References	Average SST (ºC)				Standard deviation of SST (ºC)				Number of data for averaging			
								MIS 1 (0–11 ka)	MIS 2 and deglaciation (11–29 ka)	MIS 1 (0–11 ka)	MIS 2 and deglaciation (11–29 ka)			MIS 1 (0–11 ka)	MIS 2 and deglaciation (11–29 ka)		
MD98-2181	125.83	6.30	Mg/Ca (*Globigerinoides ruber*)	Nürnberg et al. (1996)	Stott et al. (2002)			29.5	28.1	0.7	1.4			196	93		
ODP 1144	117.42	20.05	Mg/Ca (*Globigerinoides sacculifer*)	Nürnberg et al. (2000)	Wei et al. (2007)			26.3	23.9	1.0	1.1			21	47		
MD01-2421	141.78	36.03	Alkenone		Yamamoto et al. (2005)			19.1	16.0	0.8	1.4			16	61		
MD01-2420	141.82	36.07	Mg/Ca (*Globigerina bulloides*)	Mashiotta et al. (1999)	Sagawa et al. (2006)			16.3	7.2	1.9	2.5			27	41		
MD01-2412	145.00	44.53	Alkenone		Harada et al. (2006)			11.8	12.2	0.8	2.0			136	113		
ODP 882	167.58	50.35	Alkenone		Martínez-García et al. (2010)			9.0	7.4	1.6	2.4			2	4

The SSTs at all sites increased from MIS 20 to 19c, which coincides with a decreasing trend in the global benthic δ^18^O stack (Fig. 5), implying that the Pacific SSTs were subject to global glacial–interglacial changes (i.e., atmospheric CO_2_ concentration forcing and global ice volume) during Termination IX. However, SSTs dropped relatively rapidly after the peak at low-latitude ODP Site 871 and ODP Site 1146, whereas SSTs remained high even into MIS 18 at the mid-latitude CbCS and ODP 882. The mid-latitude SST pattern is similar to the EAWM record, suggesting the strong influence of EAWM variation on the mid-latitudes after MIS 19b. This contrast in SST patterns between low- and mid-latitudes suggests that the low-latitude SST is less sensitive to the EAWM and dominantly controlled by global climate drivers such as atmospheric CO_2_ concentration.

**Fig. 7:**
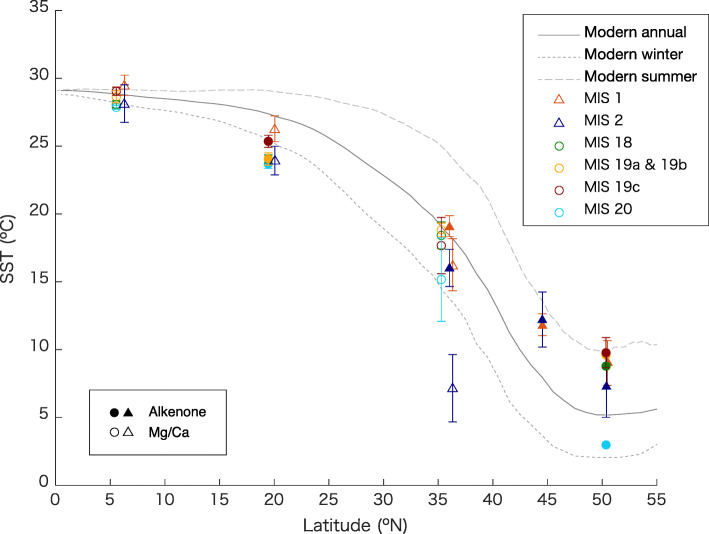
Latitudinal sea-surface temperature (SST) plot of the western North Pacific listed in Table 2 for MIS 20, 19c, 19b–19a, 18, 2, 1, and present (Locarnini et al. 2013). Filled and open symbols represent alkenone- and Mg/Ca-based SST records, respectively. Error bars are standard deviation values listed in Table 2

Comparing MIS 20–18 with MIS 2–1 provides an excellent opportunity to investigate natural climate variability and constrain predictions of future climate change. Insolation and marine records of the CbCS and the cores MD01-2421 (36° 01.4′ N, 141° 46.8′ E, 2224 m water depth) and MD01-2420 (36º 04' N, 141º 49' E, 2101 m water depth) are compared in Fig. 6. Sites MD01-2421 and MD01-2420 (hereafter MDs) are located in the Kuroshio–Oyashio mixed zone, ~ 100 km north of the CbCS with a modern annual SST ~ 1.5 °C (~ 0.4‰ on the δ^18^O scale) colder than at the latitude of the CbCS (Fig. 1b and c). Sites MDs are therefore expected to have recorded somewhat cooler conditions than the CbCS but broadly similar oceanographic information in the past. Comparisons reveal that the δ^18^O and Mg/Ca-SST records of *G*. *bulloides* in the CbCS for MIS 19 are similar to those of MDs for MIS 1 (Fig. 6 d and Fig. 7, Table 2). However, the δ^18^O_bul_ in the CbCS during MIS 20 is ~ 2‰ lower than for MDs during MIS 2 (Fig. 6d), which is not entirely accounted for in the global ice volume difference (< 0.4‰ based on Bintanja et al. 2005; Elderfield et al. 2012). The Mg/Ca-SST record also indicates an extremely low temperature (~ 7 °C) during MIS 2 (Table 2). The high δ^18^O_bul_ during MIS 2 is therefore attributed to the low SST. This is not simply explained by the latitudinal distance from the CbCS to the MD sites, as the modern winter SST gradient between the two locations is only ~ 1.5 °C (Fig. 7).

In contrast to the Mg/Ca-SST reconstruction at site MD01-2420 (Sagawa et al. 2006), alkenone-based SSTs show a drop of only 3 °C on average during MIS 2 at site MD01-2421 (Table 2) (Yamamoto et al. 2005). The SST difference between the two proxies probably stems from the seasonality, and the foraminiferal proxies likely capture information on the colder seasons unlike the alkenones as mentioned earlier. The low SSTs derived from *G*. *bulloides* therefore suggest severe winters during MIS 2. Severe cooling during MIS 2 is also suggested by the calcareous nannofossil assemblage-based temperature reconstruction (Tn), characterized by the higher abundance of *C*. *pelagicus* during MIS 2 (Aizawa et al. 2004). Although the calcareous nannofossil assemblages tend to shift dramatically within narrow transitional areas such as the Kuroshio–Oyashio mixed zone, *C*. *pelagicus* occupied ~ 60% of the subordinate taxa during MIS 2 (Aizawa et al. 2004), whereas *C*. *pelagicus pelagicus* and *C*. *pelagicus braarudii* accounted for only 40% of the subordinate taxa during MIS 20 (Kameo et al. 2020).

To understand the mechanisms underlying the significant difference between MIS 20, MIS 18, and MIS 2, we further examine the western North Pacific SST record by comparing MIS 20 and MIS 18 with MIS 2, and MIS 19 with MIS 1 (Fig. 7, Table 2). Although the SST at ODP Site 882 at 50°N indicate a dramatic cooling during MIS 20, this should be treated with caution because it is based on a single data point (Table 2). The cooling during MIS 20 is comparable to MIS 2 at low latitudes. However, the Mg/Ca proxies for MD01-2421 during MIS 2 are exceptional in the Kuroshio–Oyashio mixed zone, suggesting significantly milder winters for MIS 20 than for MIS 2. A mild winter climate, inferred from a weak EAWM, has also been presented from Chinese Loess grain-size records during MIS 20 (Hao et al. 2012; Sun et al. 2019). Hao et al. (2012) argued that glaciations during MIS 20 and 18 were initiated by very weak precessional insolation minima followed by warm summer conditions unfavorable to Northern Hemisphere ice-sheet growth at the inception of these glacial periods. As a result, ice and snow accumulation were suppressed on the Eurasian continent, leading to a weak Siberian High and associated weak EAWM winds (Hao et al. 2012). The noticeable warm SSTs during MIS 18 at the CbCS is also in line with the weak EAWM.

There is no conclusive evidence for the mean state of the global climate, including global ice volume and atmospheric CO_2_ concentration, for MIS 20. The global ice volume increased during glacials after the EMPT, but the timing of the beginning and end of the EMPT depends on the criteria used for its definition (e.g., Clark et al. 2006; Elderfield et al. 2010; Head and Gibbard 2015a). Based on bottom water δ^18^O reconstruction and its spectral power in the southwest Pacific, changes associated with the EMPT ended ~ 650 ka (Elderfield et al. 2012). A review of sea-level proxies and reconstructions for the Pleistocene (Rohling et al. 2014) has shown significant uncertainties or discrepancies depending on the regions used for the reconstructions: − 70 to − 80 m for MIS 20 based on the Mediterranean Sea (Rohling et al. 2014) and North Atlantic (Sosdian and Rosenthal 2009) but ~− 120 m based on the southwest Pacific Ocean (Elderfield et al. 2012). Thus, there is presently no conclusive evidence that clearly explains mild winter temperatures during MIS 20 from the global ice volume perspective. Nor are there reliable atmospheric CO_2_ reconstructions at high temporal resolution for MIS 20, although a transient model simulates similar CO_2_ levels to MIS 2 for MIS 20 (Willeit et al. 2019). In contrast, the mild glacial condition for MIS 18, suggested by the sea-level proxies (Elderfield et al. 2012; Rohling et al. 2014), may be associated with the warm SST at the CbCS and weak EAWM.

Concerning the climate and ocean variability during MIS 19 and MIS 18, Suganuma et al. (2018) argued that the small thermal contrast between Siberia and the northwestern Pacific Ocean and resulting weak EAWM led to the northward migration of the gyre boundary and a more stratified near-surface water column during the later part of MIS 19 after the stadial at ~772 ka. PC3 of our study shows mostly positive scores during MIS 19a and 18, inferring stratification associated with reduced nutrient supply due to less vertical mixing of the ocean. The general trend in PC3 agrees with the interpretation of Suganuma et al. (2018) for MIS 19a and 18. However, we note that the northward migration of the gyre boundary (negative scores for PC1) and the reduced nutrient state (positive scores for PC3) are not correlated on millennial-scale (stadial–interstadial oscillations) during late MIS 19a probably due to the complex effects of temperature and δ^13^C_DIC_ on foraminiferal δ^13^C at the CbCS. The monsoon climate reconstruction throughout the Pleistocene reveals that the common temporal pattern of winter monsoon climate is seen during MIS 19–18 and MIS 11–10, where insolation forcing is similar to the modern interglacial (Hao et al. 2012). In these winter monsoon records, mild winters persisted even after summer/annual climate and atmospheric CO_2_ levels declined toward the following glacial state (Hao et al. 2012). During these interglacials, the onset of winter cooling in East Asia lagged the summer/annual climate by 30–40 kyr. This pattern is also seen in the CbCS records (PC1 and related proxies) (Figs. 2, 3, and 4), likely indicating that the marine environment in the Kuroshio-Oyashio mixed zone is subject to the East Asian winter climate system for MIS 19–18.

## Conclusions

Microfossil assemblage and geochemical (δ^18^O, δ^13^C, and Mg/Ca) data collected at high temporal resolution from the CbCS have been subjected to PCA. Results indicate that the leading mode, PC1, which carries 36.3% of the total variance, likely represents oceanic conditions driven by winter climate in the western North Pacific. The temporal variations in the leading mode are closely linked to the EAWM but are also subject to the global signal. The second mode, PC2, which carries 15.4% of the total variance, is well correlated to ocean subsurface conditions in the CbCS. The leading mode is dominated by the relative contributions of the Kuroshio and Oyashio currents, as was discussed in Suganuma et al. (2018). We further interpret the leading mode to be closely related to the north–south shift of the Pacific gyre boundary, and the second mode associated with the intensity of the subtropical gyre itself. However, the strength of the subtropical gyre as represented by the subsurface temperature in the CbCS record (the second mode) will have varied independently from the latitudinal shift of the gyre boundary, especially during MIS 19c–b. Radiolarian and calcareous nannofossil assemblages are concordant, showing similar water mass types. Our analysis reveals two aspects (surface and subsurface) of the variabilities in the marine records at the CbCS associated with the global and Asian regional climate changes. Millennial-scale variations are superimposed on the long-term trend; the Kuroshio–Oyashio boundary probably shifts southwards during the stadials and northerly during the interstadials.

Glacial MIS 20 had significantly more mild winters than MIS 2. For the interglacial MIS 19 and following glacial MIS 18, the onset of winter cooling in East Asia lagged the summer/annual climate by 30–40 kyr, which is also seen in the leading mode of the CbCS marine proxies. Stratification is also suggested during MIS 19a through 18, leading to a suppressed nutrient supply due to reduced mixing.

## Supplementary Information

**Additional file 1: Fig. S1.** Sampling horizons for the Chiba composite section (CbCS).

**Additional file 2.** Text for Table S1.

**Additional file 3: Table S1.** Microfossil, Mg/Ca, δ^18^O, and δ^13^C data from the CbCS. 

**Additional file 4: Fig. S2.** SEM photographs of the planktonic foraminifer *G. bulloides* from sample ID TB2-13. The foraminiferal tests were coated with Au for SEM. a) Spiral side (3 kV, spot size = 40), b) internal test wall (15 kV, spot size = 40), c) and d) external test surface (15 kV, spot size = 40) showing spine bases.

**Additional file 5: Fig. S3.** Comparison between NMNS and KU for δ^13^C of *G. inflata* and *G. bulloides*.

**Additional file 6: Fig. S4.** Supplement figure for Ocean Drilling Program (ODP) Sites 882, 1146, and 871.

## Data Availability

The datasets supporting the conclusions of this article are included within the article and its additional files.

## Abbreviations

CbCS: Chiba composite section
MIS: Marine Isotope Stage
SST: Sea-surface temperature
AL: Aleutian low
EAWM: East Asian winter monsoon
EASM: East Asian summer monsoon
WPWP: Western Pacific warm pool
δ^13^C_inf_: δ^13^C for *G*. *inflata*
δ^13^C_bul_: δ^13^C for *G*. *bulloides*
δ^18^O_inf_: δ^18^O for *G*. *inflata*
δ^18^O_bul_: δ^18^O for *G*. *bulloides*
Δδ^18^O_inf-bul_: Difference between δ^18^O_inf_ and δ^18^O_bul_
Δδ^13^C_inf-bul_: Difference between δ^13^C_inf_ and δ^13^C_bul_
