# Supplementary figures and images for: Paleoceanography of the northwestern Pacific across the Early–Middle Pleistocene boundary (Marine Isotope Stages 20–18)
Source: Prog Earth Planet Sci. 2021 Apr 30;8(1):29. doi: 10.1186/s40645-020-00395-3 (PMC8550468; doi:10.1186/s40645-020-00395-3)

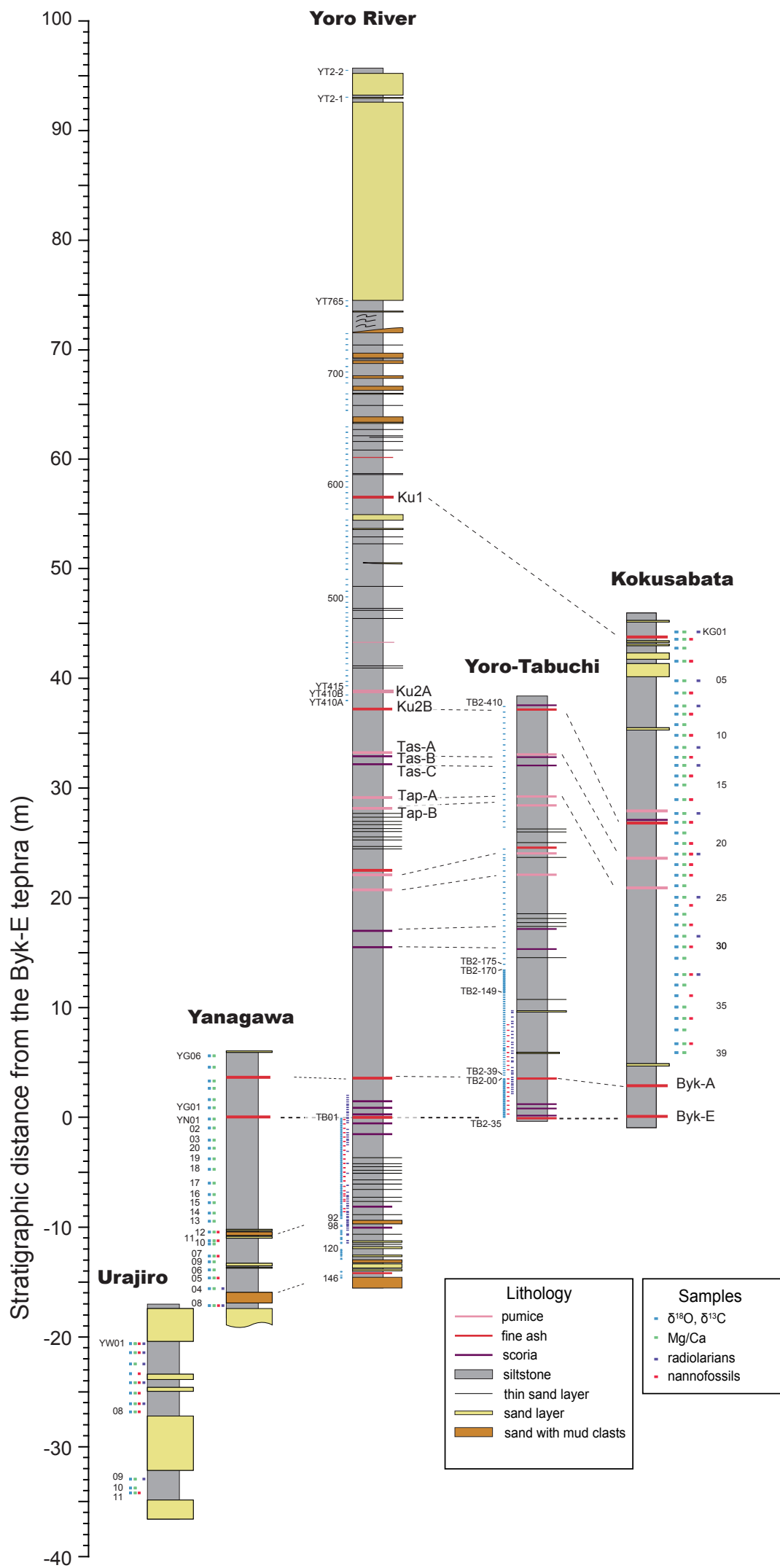

Supplement: Supplementary file 1 — Additional file 1: Fig. S1. Sampling horizons for the Chiba composite section (CbCS). [file 40645_2020_395_MOESM1_ESM.pdf]

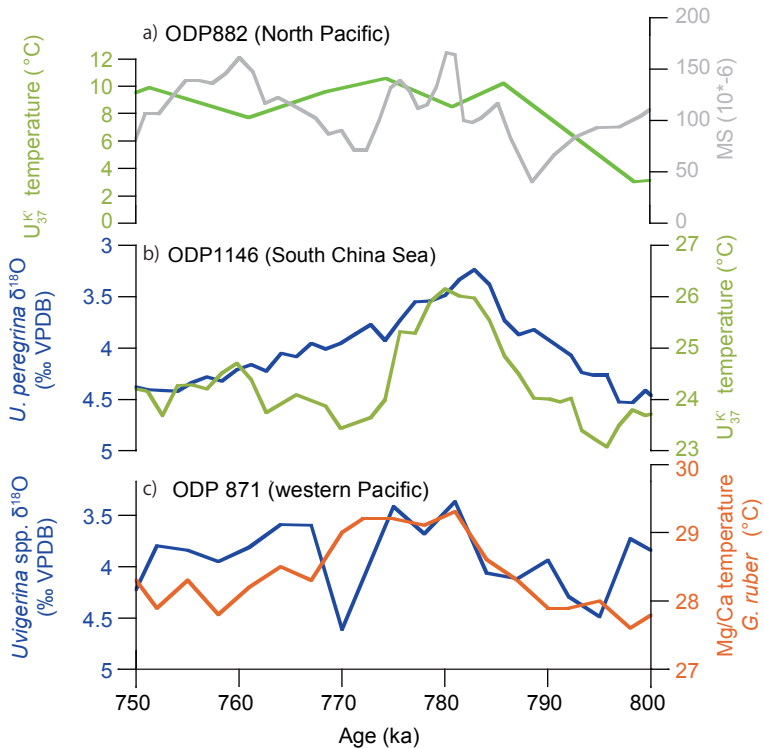

Supplement: Supplementary file 6 — Additional file 6: Fig. S4. Supplement figure for Ocean Drilling Program (ODP) Sites 882, 1146, and 871. [file 40645_2020_395_MOESM6_ESM.zip › Additional_file_S6_benthic_d18O_200831YH.pdf]
