# Supplementary material for: Paleoceanography of the northwestern Pacific across the Early–Middle Pleistocene boundary (Marine Isotope Stages 20–18)
Source: Prog Earth Planet Sci. 2021 Apr 30;8(1):29. doi: 10.1186/s40645-020-00395-3 (PMC8550468; doi:10.1186/s40645-020-00395-3)
Supplement: Supplementary file 2 — Additional file 2. Text for Table S1. [file 40645_2020_395_MOESM2_ESM.docx]

Additional file 2: Text for Table S1

The data used in this study are summarized in Additional file 3 (Table S1). For faunal and floral assemblage data, a log-ratio transformation was performed for Principal Component Analysis using the relative abundances (%) of each taxon. The positions of samples used in this study along with the lithologies of the sections from which they were collected are shown in Additional file 1 (Fig. S1). These samples are in addition to those used in Suganuma et al. (2018) and Haneda et al. (2020).

1. Radiolarian assemblage data

This includes relative abundances of six taxa newly reported in this study in addition to data already published (Suganuma et al. 2018). Zero elements in the data set were replaced by 0.105, this being half of the lowest value in the data set. The age model is based on Suganuma et al. (2018).

1. Calcareous nannofossil assemblage data

Relative abundances of six taxa are based on Suganuma et al. (2018) and Kameo et al. (2020). Zero elements in the data set were replaced by 0.5, this being half of the lowest value in the data set. A log-ratio transformation for *Florisphaera profunda* was performed separately from other species as the counting method used for this species differs from that for the other taxa. Because *F. profunda* is a dominant species in assemblages, counts for this species are based on the usual counting method of 200 specimens per slide (Suganuma et al. 2018; Kameo et al. 2020). For the other five taxa (subordinate taxa), which are rare in assemblages, additional counts using several traverses of each smear slide were made (Kameo et al. 2020). The age model is based on Suganuma et al. (2018).

1. Mg/Ca data

The Mg/Ca analysis of *G. bulloides* is newly reported in this study. The Mg/Ca values for *G. inflata* are from Suganuma et al. (2018). Their age models are based on Suganuma et al. (2018).

1. Foraminiferal oxygen and carbon isotope data

The oxygen isotope data based on Suganuma et al. (2018) and Haneda et al. (2020) were used in this study. The carbon isotope data are newly reported in this study. The oxygen isotope for PCA in this study are from the composite data presented in Haneda et al. (2020). The carbon isotope data for *G. inflata* and *G. bulloides* are newly reported in this study. The carbon isotope data of *G. bulloides* for the low resolution data set (KG, YN, YG, and YW samples) are 0.27% higher than those of the high resolution data set (Additional file 5: Fig. S3). Thus, we subtracted the offset of 0.27‰ from the KG, YN, YG, and YW samples for the carbon isotope data of *G. bulloides* (Table S1).

**References**

Haneda Y, Okada M, Kubota Y, Suganuma Y (2020) Millennial-scale hydrographic changes in the northwestern Pacific during marine isotope stage 19: Teleconnections with ice melt in the North Atlantic. Earth Planet Sci Lett 531:115936. doi: 10.1016/j.epsl.2019.115936

Kameo K, Kubota Y, Haneda Y, Suganuma Y, Okada M (2020) Calcareous nannofossil biostratigraphy of the Lower–Middle Pleistocene boundary of the GSSP, Chiba composite section in the Kokumoto Formation, Kazusa Group, central Japan, and implications for sea-surface environmental changes. Prog Earth Planet Sci 7:36. doi: 10.1186/s40645-020-00355-x

Suganuma Y, Haneda Y, Kameo K, Kubota Y, Hayashi H, Itaki T, Okuda M, Head MJ, Sugaya M, Nakazato H, Igarashi A, Shikoku K, Hongo M, Watanabe M, Satoguchi Y, Takeshita Y, Nishida N, Izumi K, Kawamura K, Kawamata M, Okuno J, Yoshida T, Ogitsu I, Yabusaki H, Okada M (2018) Paleoclimatic and paleoceanographic records through Marine Isotope Stage 19 at the Chiba composite section, central Japan: A key reference for the Early–Middle Pleistocene Subseries boundary. Quat Sci Rev 191:406–430. doi: 10.1016/j.quascirev.2018.04.022
