# Supplementary material for: Paleoceanography of the northwestern Pacific across the Early–Middle Pleistocene boundary (Marine Isotope Stages 20–18)
Source: Prog Earth Planet Sci. 2021 Apr 30;8(1):29. doi: 10.1186/s40645-020-00395-3 (PMC8550468; doi:10.1186/s40645-020-00395-3)
Supplement: Supplementary file 4 — Additional file 4: Fig. S2. SEM photographs of the planktonic foraminifer G. bulloides from sample ID TB2-13. The foraminiferal tests were coated with Au for SEM. a) Spiral side (3 kV, spot size = 40), b) internal test wall (15 kV, spot size = 40), c) and d) external test surface (15 kV, spot size = 40) showing spine bases. [file 40645_2020_395_MOESM4_ESM.docx]

Additional file S4: Fig. S2

SEM photographs of the planktonic foraminifer *G. bulloides*

Fig. S2.


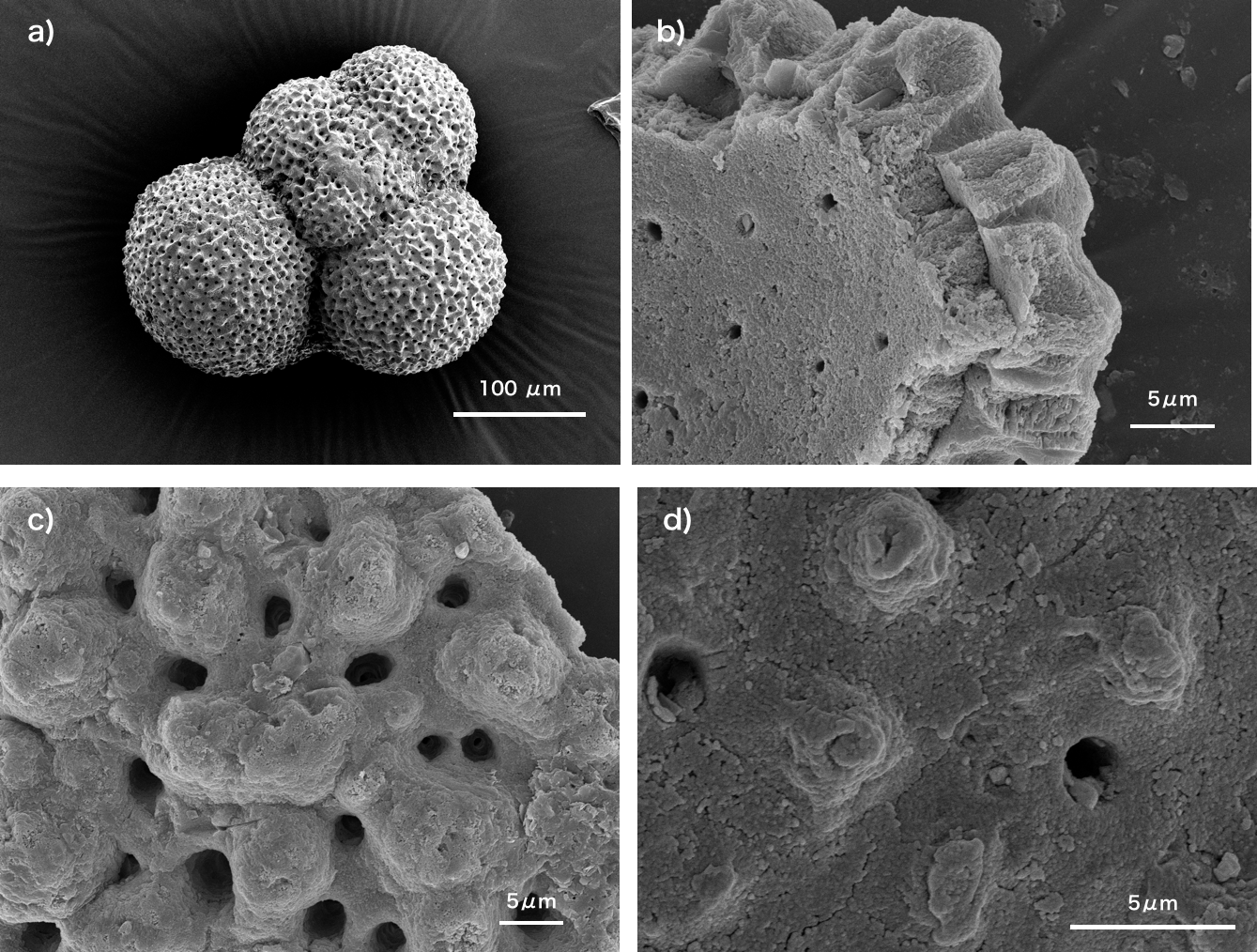


Fig. S2. SEM photographs of the planktonic foraminifer *G. bulloides* from sample ID TB2-13. The foraminiferal tests were coated with Au for SEM. a) Spiral side (3 kV, spot size = 40), b) internal test wall (15 kV, spot size = 40), c) and d) external test surface (15 kV, spot size = 40) showing spine bases.
