# Supplementary material for: Paleoceanography of the northwestern Pacific across the Early–Middle Pleistocene boundary (Marine Isotope Stages 20–18)
Source: Prog Earth Planet Sci. 2021 Apr 30;8(1):29. doi: 10.1186/s40645-020-00395-3 (PMC8550468; doi:10.1186/s40645-020-00395-3)
Supplement: Supplementary file 5 — Additional file 5: Fig. S3. Comparison between NMNS and KU for δ13C of G. inflata and G. bulloides. [file 40645_2020_395_MOESM5_ESM.pdf]

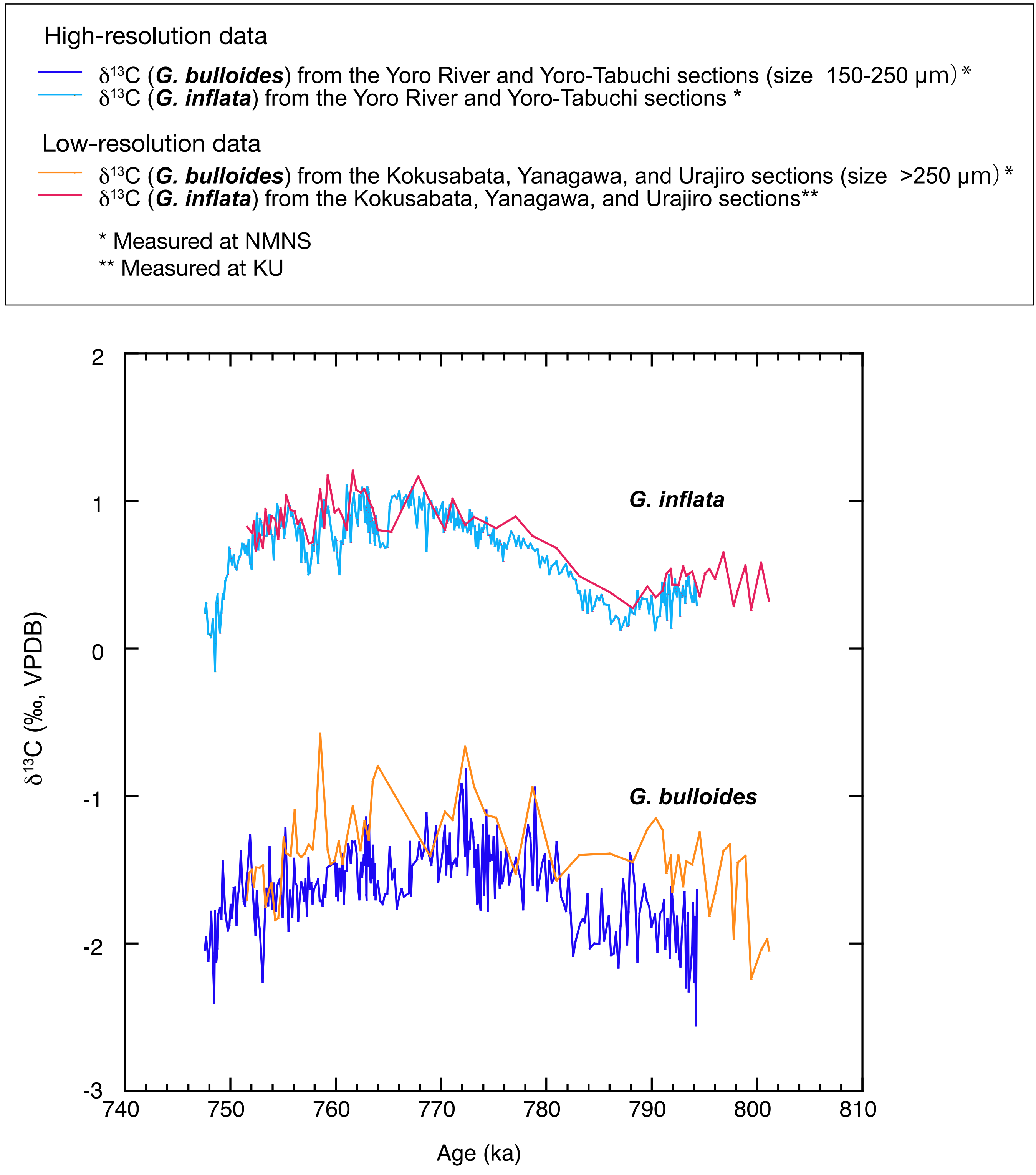
High-resolution data
δ13C (G. bulloides) from the Yoro River and Yoro-Tabuchi sections (size 150-250 μm) *
δ13C (G. inflata) from the Yoro River and Yoro-Tabuchi sections *
Low-resolution data
δ13C (G. bulloides) from the Kokusabata, Yanagawa, and Urajiro sections (size >250 μm) *
δ13C (G. inflata) from the Kokusabata, Yanagawa, and Urajiro sections**
* Measured at NMNS
** Measured at KU
G. inflata
G. bulloides
δ13C (‰, VPDB)
Age (ka)
2
1
0
-1
-2
-3
740
750
760
770
780
790
800
810
