# Supplementary material for: Paleoceanography of the northwestern Pacific across the Early–Middle Pleistocene boundary (Marine Isotope Stages 20–18)
Source: Prog Earth Planet Sci. 2021 Apr 30;8(1):29. doi: 10.1186/s40645-020-00395-3 (PMC8550468; doi:10.1186/s40645-020-00395-3)
Supplement: Supplementary file 6 — Additional file 6: Fig. S4. Supplement figure for Ocean Drilling Program (ODP) Sites 882, 1146, and 871. [file 40645_2020_395_MOESM6_ESM.zip › Additional_file_6_ESM.docx]

Additional file 6: Fig. S4. Supplement figure for Ocean Drilling Program (ODP) Sites 882, 1146, and 871.

Fig. S4.

Fig. S4. Supplement figure for a) U temperature (Martínez-García et al. 2010) and magnetic susceptibility (Rae and Basov 2005) records of ODP 882, b) U temperature and benthic δ^18^O of ODP Site 1146 (Herbert et al. 2010), and c) Mg/Ca temperature and benthic δ^18^O of ODP Site 871 (Dyez and Ravelo 2014).

**References**

Dyez KA, Ravelo AC (2014) Dynamical changes in the tropical Pacific warm pool and zonal SST gradient during the Pleistocene. Geophys Res Lett 41:7626–7633. doi: 10.1002/2014GL061639

Herbert TD, Peterson LC, Lawrence KT, Liu Z (2010) Tropical ocean temperatures over the past 3.5 million years. Science 328:1530–1534. doi: 10.1126/science.1185435

Martínez-García A, Rosell-Mele A, McClymont EL, Gersonde R, Haug GH (2010) Subpolar link to the emergence of the modern equatorial Pacific cold tongue. Science 328:1550–1553. doi: 10.1126/science.1184480

Rea DK, Basov IA (2005) Shipboard Scientific Party: Magnetic susceptibility on ODP Hole 145-882B. PANGAEA, <https://doi.org/10.1594/PANGAEA.265695>
